# From artery to memory: a comparative review of vascular cognitive impairment surgical models

**DOI:** 10.3389/fnagi.2026.1861974

**Published:** 2026-06-10

**Authors:** Micayla M. Kane, Sydney E. Sneed, Erin E. Kaiser

**Affiliations:** 1 Regenerative Bioscience Center, University of Georgia, Athens, GA, United States; 2 Department of Animal and Dairy Science, College of Agricultural and Environmental Sciences, University of Georgia, Athens, GA, United States; 3 Neuroscience Program, Biomedical and Health Sciences Institute, University of Georgia, Athens, GA, United States

**Keywords:** cerebral hypoperfusion, cognitive decline, common carotid artery, neurodegenerative disease, surgical models, vascular cognitive impairment, white matter

## Abstract

Vascular cognitive impairment (VCI) encompasses a spectrum of cerebrovascular diseases ranging from mild clinical cognitive impairment to advanced vascular dementia and is recognized as a major contributor to the global dementia burden. Frequently coexisting with Alzheimer’s Disease (AD), VCI represents a complex, mixed-pathology neurodegenerative process driven by chronic cerebral hypoperfusion (CCH), white matter (WM) injury and volume loss, neurovascular dysfunction, and progressive cognitive decline. While numerous animal models have been developed to characterize the underlying mechanisms and identify therapeutic targets, the field is presently limited by the absence of a distinct framework to guide model selection based on unique pathophysiological features of recently delineated VCI subtypes. Surgical VCI models, including transient and permanent occlusion, stenosis, or gradual occlusion approaches, differ substantially in the duration of ischemic injury, severity of hypoperfusion, and mechanism of cerebral blood flow (CBF) reductions, generating diverse downstream effects on cerebral tissue damage, neuroinflammation, neurometabolic dysfunction, functional integrity, and, ultimately, memory function. No single model completely captures the heterogeneity of VCI pathology; however, each selectively captures unique aspects of disease subtypes. As such, this review aims to establish a clear, pathophysiology-driven framework to guide the selection of appropriate surgical VCI models for investigating specific VCI subtypes. To do so, we evaluate common models of carotid artery manipulation, integrating histological, neuroenergetic, and cognitive outcomes with clinically relevant imaging and patient data. This review provides practical guidance for model selection, enhancing the specificity and translational relevance of preclinical VCI investigation.

## 1 Introduction

Vascular cognitive impairment (VCI) is a chronic and degenerative cerebrovascular disease characterized by white matter (WM) lesions and degeneration, microinfarcts, small vessel disease, and progressive cognitive dysfunction (Corriveau et al., 2016). VCI encompasses a broad spectrum of cerebrovascular disease, from mild cognitive dysfunction to clinically diagnosed Alzheimer’s Disease (AD) and vascular dementia. The World Health Organization broadly identifies dementia as the 7th leading cause of death globally, with 57 million affected individuals in 2021 and an expected annual increase by 10 million (World Health Organization, 2025). While the most prevalent form of dementia is AD, contributing to 60–70% of cases, VCI has more recently been identified as both an independent form of dementia and, perhaps more significantly, a concomitant contributor to mixed-pathology dementia paradigms (World Health Organization, 2025). A growing body of literature increasingly supports a mixed pathological mechanism of VCI and AD, with primary overlapping features including cerebral amyloid angiopathy, small vessel disease, endothelial dysfunction, neuroinflammation, and diffuse WM changes which synergistically drive disease onset and progression (Corriveau et al., 2016; Gooch and Wilcock, 2016).

Clinically, VCI ranges from subtle impairments in executive function and processing speed to advanced vascular dementia characterized by significant cognitive decline, gait dysfunction, neuropsychiatric impairment, and progressive loss of functional independence (Cavanagh and Paulsen, 2024; Beauchet et al., 2016; Chen et al., 1998; Montero-Odasso et al., 2012; Moretti et al., 2011; Perez et al., 1975; Sakurai et al., 2020; Sultzer et al., 1993; Temedda et al., 2025). While AD routinely presents with memory and language deficits, VCI patients more prominently experience frontal-subcortical-dependent executive function deficits such as reduced processing speed and impaired executive function (Kalaria, 2016; Chen et al., 1998). Discrepancies in symptom presentation, despite frequent pathophysiological overlap, likely stem from vascular insufficiency of selectively vulnerable WM tracts (Kalaria, 2016). Neuroimaging of VCI patients commonly report WM hyperintensities, lacunar and silent infarcts, altered perivascular space, cortical and subcortical atrophy, and diffuse reductions in vascular perfusion (Clancy et al., 2024; Heiss et al., 2016; Chouliaras and O’Brien, 2023; Lowry et al., 2021). Interestingly, the severity of imaging abnormalities may not consistently correlate with symptom burden or presentation of cognitive impairment (Mok, 2023). Accordingly, VCI is best understood as a multifactorial neurodegenerative disease in which chronic cerebral hypoperfusion (CCH) accelerates neuropathological degeneration and cognitive decline.

The interaction of cerebral blood flow (CBF) and neural cells is tightly controlled by the neurovascular unit, a network of interactive neurons, astrocytes, and microglia, the dysregulation of which is central to VCI pathogenesis (Rundek et al., 2022). As CBF decreases in the pathologic brain, the nutrient supply is decreased, intercellular signaling is impeded, debris clearance mechanisms are inhibited, and overall cell dysregulation and death become prominent. Chronic hypoperfusion contributes to blood brain barrier (BBB) breakdown, glial activation, axonal degeneration, and degradation of vulnerable WM tracts, which comprise the most consistent radiographic and histopathological hallmarks in clinical patients with diagnosed VCI and vascular dementia. Concomitantly with such cellular changes, neurotransmitter and neurometabolic homeostasis is interrupted in the VCI brain. Alterations in various neurotransmitters have been linked to clinical symptoms associated with VCI and vascular dementia such as cognitive decline secondary to cholinergic and glutamatergic deficits, EEG brainwave pattern changes associated with excitatory and inhibitory changes, and psychiatric symptoms related to monoamine neurotransmission modifications (Teleanu et al., 2022).

Despite growing recognition of vascular contributions to cognitive decline and dementia pathogenesis, effective therapies remain limited and incomplete, challenged by the substantial heterogeneity of VCI pathophysiology, the frequent coexistence of cerebrovascular and neurodegenerative disease processes, and the limited translational efficacy of existing experimental paradigms (Corriveau et al., 2016; Iadecola et al., 2019). As such, numerous animal models have been developed to better characterize key features of CCH, ischemic injury cascades, neuroinflammation, and progressive cognitive and functional decline. However, these models vary considerably in their replication of temporal progression, specific tissue vulnerability, hemodynamic adaptations, and neuropathological complexity consistent with clinical disease.

**Figure 1:**
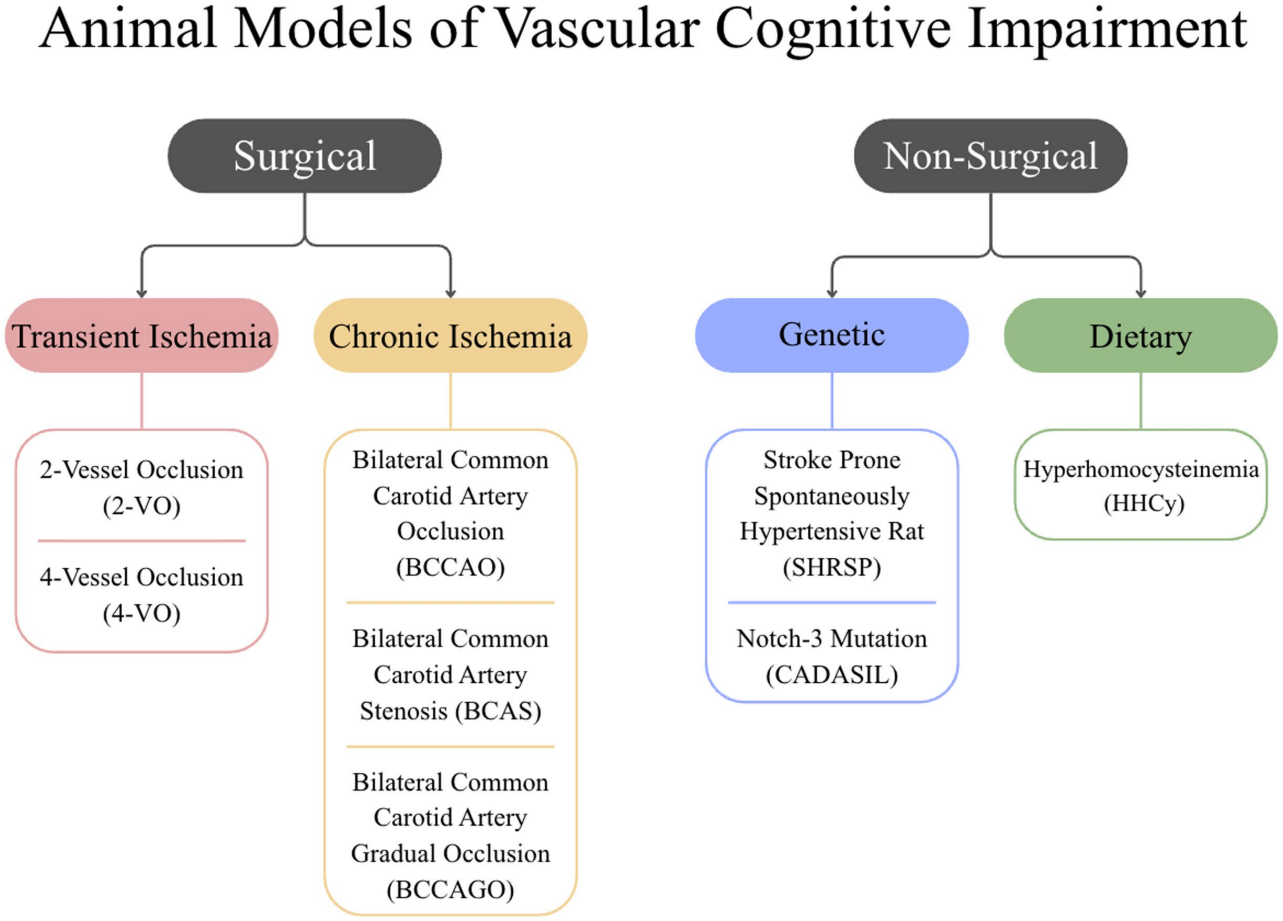
Classification of surgical and non-surgical vascular cognitive impairment (VCI) animal models. VCI surgical models can be considered transient or chronic, where the former causes brief vascular injury and the latter produces progressive or sustained reductions in cerebral blood flow (CBF). Transient global ischemia models include temporary two-vessel occlusion (2-VO) and four-vessel occlusion (4-VO) models. Chronic global ischemia models include bilateral common carotid artery occlusion (BCCAO), bilateral common carotid artery stenosis (BCAS), and bilateral common carotid artery gradual occlusion (BCCAGO). Non-surgical models utilize genetic manipulation which includes selective breeding for the stroke prone spontaneously hypertensive rat and knockout Notch-3 mutations to induce cerebral autosomal dominant arteriopathy with subcortical infarcts and leukoencephalopathy as well as the dietary induction of hyperhomocysteinemia.

While numerous models surgically induce global transient or CCH in rodents, other non-surgical methods and large animal hypoperfusion models have been used with unique limitations (Figure 1). Common non-surgical models include dietary manipulation to induce hyperhomocysteinemia, cerebral amyloid angiopathy induction, genetic manipulation to trigger cerebral autosomal dominant arteriopathy with subcortical infarcts and leukoencephalopathy (CADASIL) pathology, and replication of post-stroke vasculopathy via stroke prone spontaneously hypertensive rats (Arvanitakis et al., 2016; Sudduth et al., 2017; Tuo et al., 2021; Hainsworth et al., 2017). Surgically induced VCI models most successfully recapitulate the heterogeneity of physiological and cognitive outcomes consequential to CCH with greater replicability and predictability than the non-surgical methods (Gooch and Wilcock, 2016; Sudduth et al., 2017; Tuo et al., 2021). Rodent VCI models are the most prevalent, with limited literature available utilizing large animal models due to financial, practical, and ethical constraints (Hainsworth et al., 2017; Washida et al., 2019). Ovine and caprine models of middle cerebral artery occlusion most closely replicate ischemic stroke pathology (Boltze et al., 2008; Wells et al., 2012). Non-human primate models offer greater translatability of cognitive and neuroimaging outcomes, but due to the complexity of surgical induction techniques, most models evaluate non-surgical dementia comorbidities (Hainsworth et al., 2017; Li et al., 2019). Further investigations are warranted in large animal models to improve the translatability of research outcomes from VCI modeling.

Experimental VCI surgical models primarily include transient ischemic models, including two-vessel occlusion (2-VO) and four-vessel occlusion (4-VO) in addition to chronic hypoperfusion models such as bilateral common carotid artery occlusion (BCCAO), bilateral common carotid artery stenosis (BCAS), and bilateral common carotid artery gradual occlusion (BCCAGO). Each model generates broad overlapping pathophysiology while retaining distinct aspects relative to acute or progressive cerebrovascular insufficiency (Goto et al., 1990; Gregory et al., 2001; Hartman et al., 2005; Heim et al., 2000; Irie et al., 2002; Iwasaki et al., 1996; Petito et al., 1998; Pulsinelli and Brierley, 1979; Schmidt-Kastner et al., 1989; Sugawara et al., 1999; Chung et al., 2002; Ihara and Tomimoto, 2011; Khan et al., 2015). These surgical VCI models have effectively reproduced key pathophysiological features of clinical VCI, such as impaired CBF, development of WM lesions, increased neuronal inflammation, BBB disruption, and working memory deficits without extraneous pathologies that more closely align with stroke or acute traumatic injury. Achieving a CCH state through occlusion or stenosis of the common carotid arteries has become a reliable model for evaluating consequent brain ischemia pathologies. Modern VCI surgical models clearly demonstrate drastic and persistent reductions in CBF, disrupted glucose metabolism, neurotransmitter imbalance, and neurodegeneration which align with clinical VCI pathophysiology, allowing researchers to utilize these models to pursue novel diagnostic and therapeutic measures.

There are numerous reviews available which thoroughly analyze the complex pathophysiology underlying the clinical symptoms of VCI as a spectral disorder, synthesized from both clinical data and animal models (Rundek et al., 2022; Tao et al., 2025; Clancy et al., 2024; Lennon and Sachdev, 2026; Yang, 2025). Surgical animal models of VCI have emerged as a highly controlled and reproducible method for inducing cerebral hypoperfusion and generating clinically relevant tissue damage and cognitive deficits. This review critically evaluates currently utilized surgical VCI models, emphasizing their translational relevance to clinical VCI subtypes. Particular focus is placed on pathological outcomes such as WM degeneration, BBB disruption, neuroinflammation, neuronal population loss, neuroenergetic dysfunction, and cognitive impairment across acute and chronic timepoints in both acute and chronic models of VCI. Experimental outcomes are integrated with clinically relevant neuroimaging, histopathological, and functional patient data to identify key areas of convergence and divergence between preclinical models and human VCI pathology. Through comparative analysis of transient or chronic occlusive, stenotic, or gradual hypoperfusion models, this review aims to clarify which surgical approaches most effectively recapitulate clinically relevant VCI disease subtypes while highlighting persistent limitations, translational gaps, and opportunities for future model refinement or generation of novel paradigms.

### 1.1 Search strategy and selection criteria

A structured literature search was conducted to identify studies describing surgical animal models of VCI. The literature selected for this review was obtained via PubMed, Google Scholar, ScienceDirect, Springer, Frontiers, and Nature. The search was limited to articles published between 1979 and 2025, reflecting the timeline of VCI surgical model development, characterization, and expansion. Reference lists of relevant articles were also manually screened to identify additional studies not captured through database searches.

The following keywords were used both independently and in combination to identify and refine relevant articles: vascular cognitive impairment, VCI, neurovascular unit, neurotransmitter, neuroenergetic, neurometabolic, animal models, surgical models, common carotid artery ligation surgery, common carotid artery stenosis surgery, gradual occlusion common carotid surgery, chronic cerebral hypoperfusion, vascular dementia, neurodegenerative disease, cognitive impairment, common carotid occlusion, common carotid stenosis, BCCAO, BCAS, 2-VO, 4-VO, transient occlusion, ameroid constrictor, ameroid ring, casein constrictor, magnetic resonance arteriography, and magnetic resonance imaging.

Additional terms were applied to capture species-specific studies, including pig, porcine, swine, sheep, ovine, goat, caprine, non-human primate, large animal model, mouse, murine, rat, rodent, small animal model. Comparative clinical VCI literature was applied to this review, and relevant papers were identified with the following keywords: clinical VCI, vascular dementia, VCI patient, human vascular cognitive disease, cerebral small vessel disease, cSVD, post-stroke dementia, PSD, multi-infarct dementia, MID, subcortical ischemic vascular dementia, SIVD, vascular contribution to cognitive impairment and dementia, VCID, neuropsychological impairment, cognitive decline.

Studies were included if they described surgically induced cerebral hypoperfusion models (acute or chronic) relevant to VCI and reported histological, imaging, physiological, cognitive, or functional outcomes following model induction. Both rodent and large animal studies were eligible for inclusion, and all studies were peer-reviewed primary research articles. Studies were excluded from the review which modeled VCI through dietary or genetic manipulation without a surgical component. Stroke paradigm papers were excluded if they did not assess WM or cognitive outcomes.

Titles and abstracts were initially screened to identify potentially relevant articles, followed by full-text review to confirm both article relevance to surgical VCI models and the presence of experimental endpoints. From eligible studies, data on model type, species, method of vascular manipulation, CBF reduction severity, mortality rate, neuropathological and neurometabolic findings, and cognitive outcomes were narratively synthesized and compared for experimental strengths, limitations, and translational relevance.

## 2 Vascular cognitive impairment surgical animal models

### 2.1 Transient global ischemia

#### 2.1.1 Two-vessel occlusion with reperfusion

The two-vessel occlusion (2-VO) rat model is one of the earliest VCI surgical animal models in which transient global cerebral hypoperfusion is achieved through temporary restriction of blood flow bilaterally through the common carotid arteries. The common carotid arteries are identified and isolated from the surrounding cervical tissue through a ventral incision. Metal atraumatic arterial clamps are placed around both carotid arteries and occlusion is maintained for 8–60 min intervals that may be repeated (Wahul et al., 2018; Walker and Rosenberg, 2010; Hartman et al., 2005). Reperfusion is permitted to occur from 1 h to 7 days after removal of ligation clips prior to sacrifice (Ábrahám and Lázár, 2000; Walker and Rosenberg, 2010; Wahul et al., 2018; Hartman et al., 2005; Irie et al., 2002; Heim et al., 2000; Onken et al., 2012).

This model is procedurally similar to Longa’s ischemic stroke rodent model, in which the common carotid artery is transiently occluded and the external carotid artery ligated (Justić et al., 2022; Li et al., 2023; Li et al., 2013; Longa et al., 1989). The transient occlusion of the common carotid arteries in 2-VO and the external carotids in an ischemic stroke model both produce drastic reductions in global CBF (Duncombe et al., 2017; Wei et al., 2008). During the 2-VO occlusive phase, CBF was reduced to 11.2 ± 1.1% of baseline (i.e., pre-operative) flow (Walker and Rosenberg, 2010; Duncombe et al., 2017; Wei et al., 2008). Immediately following ligation clip removal, CBF was restored to 44.9 ± 6.3% of baseline (Walker and Rosenberg, 2010). In comparison, CBF decreased to 17.9 ± 5.0% immediately following ischemic stroke modeling and remained comparably depressed, at or below 50% baseline at 30–120 min post-ischemia (Taguchi et al., 2010; Li et al., 2013). Interestingly, CBF specifically within cortical branches was generally restored by 120 min of post-stroke reperfusion, but striatal CBF typically remained depressed (Kleinschnitz et al., 2015; Unekawa et al., 2024). 2-VO models have demonstrated preferential CBF reductions in the hippocampus and striatum which correlate most robustly with neuroenergetic alteration and WM lesioning (Heim et al., 2000). Based on the magnitude and rapidity of CBF reduction and restitution, the 2-VO model is best positioned as an experimental paradigm of acute global ischemic and reperfusion injury, closely aligning with downstream mechanisms underlying post-stroke dementia (PSD).

Histologically, 2-VO induces several deficits across neural cell types through pro-inflammatory mediators and enzymatic dysregulation. Pyramidal CA1 neuron populations decreased by up to 50% globally following 2-VO with reperfusion (Hartman et al., 2005; Sugawara et al., 1999; Wei et al., 2008). By 3 days post-operatively, a significant portion of CA1 neurons were morphologically abnormal or displayed pyknotic nuclei (Sugawara et al., 1999). Viable and metabolically active neural cell populations were reduced in both the striatum and cortex acutely (1 day) after 2-VO. However, reductions in hippocampal neurons did not appear with equal severity until later timepoints (7 days), indicating that apoptosis and degeneration of cerebral tissue is both compartment and time specific following ischemia and reperfusion with the 2-VO method (Wahul et al., 2018).

**Figure 2:**
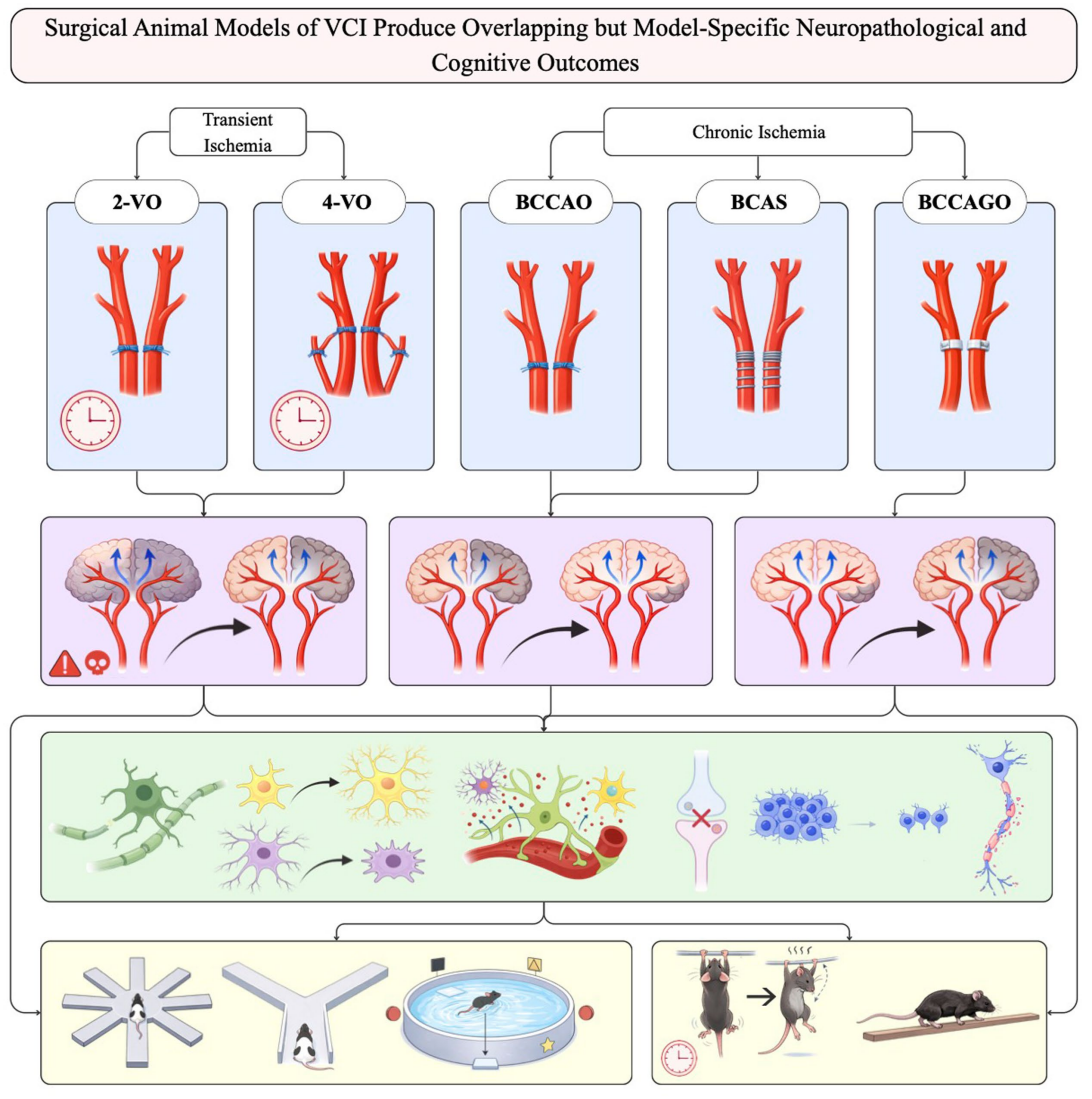
Comparative overview of commonly used surgical animal models of vascular cognitive impairment (VCI) and their associated neuropathological and functional outcomes. Models are organized by transient or chronic ischemia, including transient two-vessel occlusion (2-VO) and four-vessel occlusion (4-VO) and chronic bilateral common carotid artery occlusion (BCCAO), bilateral common carotid artery stenosis (BCAS), and bilateral common carotid artery gradual occlusion (BCCAGO). Transient models produce acute reductions in blood flow followed by reperfusion, whereas chronic models generate sustained hypoperfusion. Despite model-specific differences in ischemic severity and temporal progression, these paradigms produce overlapping neuropathological figures including white matter injury, glial activation, astrocytosis, neuroinflammation, blood–brain barrier dysfunction, synaptic impairment, neuronal loss, and neurovascular uncoupling. These pathological changes contribute to deficits in learning, working memory, executive function, and sensorimotor performance, evaluated using cognitive behavioral assays such as the Morris water maze, radial arm maze and Y-maze. The BCCAGO model notably produces deficits in motor coordination tasks such as the balance beam and wire hang test.

Microglia play a complex, dual-purpose role in the pathogenesis of VCI, where they act acutely to mitigate damage from ischemic injury, but chronically contribute to numerous degenerative processes mediated by prolonged neuroinflammation. In the 2-VO model, mild microglial activation occurred immediately following reperfusion in groups with a 10 and 15 min ischemic interval; whereas 20-min occlusion models exhibited robust microglial activation less than 1 h post-reperfusion (Ábrahám and Lázár, 2000). Extended periods (up to 72 h) after 2-VO, microglial upregulation regressed to categorically mild and moderate intensities; however, intense Iba-1 + populations did persist at 3 and 7 days following transient ischemia (Figure 2) (Ábrahám and Lázár, 2000; Walker and Rosenberg, 2010). Immediate post-operative microglial upregulation indicates an urgent neuroinflammatory response to acute ischemic injury, which is expected given the role of glia in pro-inflammatory injury mitigation. While the acute microglial response is vital for survival and recovery, chronic activation and neuroinflammation contributes to the cyclical degradation of healthy neural tissue and resultant neurodegenerative disease (Alavez-Rubio and Juarez-Cedillo, 2024; Chen et al., 2018). Observed ramified morphological shifts suggest a more chronic microglial involvement in deleterious phagocytosis and synaptic pruning following ischemic injury (Lituma et al., 2021). This chronic reperfusion injury phase mediated by neuroinflammation represents a prime target for potential therapies, where a reversible amount of microglial activation could be controlled to reduce inflammation and subsequent tissue damage; however, the 2-VO model limitations constrain its utility to that goal.

During acute neural inflammation, mixed metalloproteinases (MMPs) are abundant and play a pivotal role in endothelial tight junction cleavage and subsequent BBB breakdown. The synchronous perpetuation of these MMPs (namely, MMP-2 and MMP-9) contributes to eventual BBB disruption in VCI pathophysiology, yet the brevity of 2-VO studies limits evaluation of BBB integrity at chronic timepoints following restitution of blood flow (Xu et al., 2022). MMP-2 is largely responsible for the enzymatic degradation of myelin-basic protein (MBP) and endothelial tight junction proteins. When persistently upregulated, the mechanistic action of MMP-2 detrimentally contributes to demyelination and BBB disruption, allowing larger reactive oxygen species (ROS) and pro-inflammatory cytokines to degrade cerebral tissue (Walker and Rosenberg, 2010; Madigan et al., 2016; Sweeney et al., 2018). Lakhan et al. asserts that MMP-9 is more closely associated with direct BBB degradation through cleavage of both tight junction proteins and basal membrane proteins (Lakhan et al., 2013). Studies show that up to 7 days following reperfusion in a 2-VO model, MMP-2 was significantly elevated despite insignificant changes in MMP-9, suggesting that increased WM damage at these timepoints may be due to enzymatic activity of MMP-2 rather than active BBB damage through MMP-9 endothelial damage (Walker and Rosenberg, 2010; Lakhan et al., 2013). However, it has been shown that MMPs are involved in the degradation of MBP, thus contributing to demyelination and WM lesion formation (Walker and Rosenberg, 2010). Hemorrhagic transformation (transition of a brain infarct to an area of hemorrhage) is identified in clinical autopsy of post-stroke patients, implicating MMP-mediated BBB disruption in both clinical PSD disease and 2-VO modeling (Kawa et al., 2025; Lakhan et al., 2013). Importantly, hemorrhagic transformation is apparent within 2–14 days following ischemic stroke and can therefore be visualized in 2-VO models which extend beyond 7 days. MMP-mediated neural disruption may be studied using the 2-VO model; however, additional contributors to BBB breakdown and myelin damage must be considered within the short post-reperfusion time course.

The 2-VO model only mimics the chronic ischemic conditions of VCI acutely, limiting its utility for longitudinal changes in WM following CCH; however, studies have shown that WM damage is still detectable. Specifically, viable oligodendrocyte loss is implicated in both de- and dysmyelination. Oligodendrocyte populations were reduced in WM tracts at both 3 and 7 days post 2-VO, the loss of which contributed directly to downstream axonal and synaptic damage (Walker and Rosenberg, 2010). While precise mechanisms remain challenging to ascertain, ischemic-induced oligodendrocyte apoptosis may play a critical role in the delayed development of chronic inflammation and WM injury (Walker and Rosenberg, 2010). In addition to direct oligodendrocyte loss, astrocytosis was notably increased in GFAP+ hypoxic WM at 1–7 days post-procedure (Walker and Rosenberg, 2010). This hypertrophic astrocyte response following ischemic and reperfusion injury has been shown to contribute to myelin debris accumulation and inhibition of myelin homeostatic pathways (LCN2/LRP1) (Yang et al., 2023). Given the acute sacrifice timepoints (up to 7 days) of 2-VO models, chronic WM damage in response to proliferative neuroinflammation is difficult to determine.

Imbalances in neurotransmitter release secondary to acute ischemia (such as dopamine, GABA, and glutamate) may delineate a relationship between ischemic-induced excitotoxicity, neuronal apoptotic cascades, oligodendrocyte and myelin loss, and functional memory regression (Heim et al., 2000; Matute et al., 2007). Glutamate, specifically, contributes to cyclical excitotoxicity in conjunction with an imbalanced inward cellular flow of calcium (Wang et al., 2024). Excessive circulating glutamate overstimulates NMDA receptors, triggering apoptotic neuronal cell death pathways, contributing to downstream neuronal density reductions in both 2-VO VCI models and ischemic stroke models (Lai et al., 2014; Wang et al., 2024; Zhang and Bhavnani, 2006; Iwasaki et al., 1996). Furthermore, Wahul et al. reported decreased expression of CA1 and CA2 hippocampal NMDA receptors, suggesting a mechanism underlying long-term memory deficits in the 2VO VCI model (Wahul et al., 2018). Acute neuronal cell death contributes to subsequent cognitive decline. Accordingly, the 2-VO model may prove particularly useful for investigating neurotransmitter-based therapeutic targets in post-stroke dementia due to the linear relationship between acute neurotransmitter imbalance following ischemic stroke and chronic cognitive decline (Wang et al., 2024).

Heim et al. suggests a positive correlation between extracellular neurotransmitter concentration differences and progressive cognitive deficiency in a 2-VO rat model (Heim et al., 2000). Robust discrepancies in Morris water maze performance indicated spatial working and reference memory impairment by 7 days following a 10 min 2-VO ischemic injury in rats (Hartman et al., 2005; Wahul et al., 2018; Heim et al., 2000). Latency to escape increased consistently from 6 to 13 months post-operatively, indicating regression of learning and motor skills (Heim et al., 2000). Additionally, 2-VO rats exhibited worsened outcomes of the 8-arm radial maze than sham animals, taking more days to attain satisfactory training acquisition criterion and committing more errors during testing (Hartman et al., 2005). Discrepancies in the 8-arm radial maze correlate 2-VO with impaired spatial working memory and spatial learning. The correlation between spatial memory deficits and histological changes do recapitulate some VCI patient outcomes, particularly acute histological changes and subsequent cognitive deficiencies (Taguchi et al., 2010; Li et al., 2013; Walker and Rosenberg, 2010).

##### 2.1.1.1 2-VO model considerations

Transient ischemia models permitting reperfusion may provide greater insight into post-stroke or transient ischemic attack pathology rather than mild to moderate VCI, uniquely characterized by chronic and progressive disease mechanisms with limited recovery. Sequential removal of atraumatic arterial clamps reduces the risk of reperfusion injury, a phenomenon more characteristic of acute ischemic stroke than VCI, but still relevant to chronic post-stroke dementia pathophysiology. The possibility of reperfusion injury in the 2-VO model may be of interest for cohorts surviving greater than 7 days, as oxidative stress, neuroinflammation, and microvascular obstruction may inform prolonged outcomes (Khan et al., 2025). An adaptation of the 2-VO model to greater than 6-month survivability may better inform specific post-stroke cognitive impairment (Jiménez-Ruiz et al., 2024; Mijajlović et al., 2017). While the 2-VO model acutely and severely reduces CBF, cerebral tissue does not experience the sustained hypoxia that generates VCI-associated neurometabolic deficits (Eklöf and Siesjö, 1972; Xiang et al., 2021; Lourenço et al., 2015). Transient hypoperfusion models do successfully recapitulate a multitude of acute post-ischemic outcomes, such as diffuse WM lesions, neuroinflammation, gliosis, neuronal loss, and cognitive deficits (Figure 2). These models, however, only evaluate acute timepoints, which do not accurately reflect the progressively degenerative nature of VCI. Additionally, the transient ischemic injury due to reperfusion poorly replicates the gradual deterioration of cerebral small vessels that underlies the majority of mixed-pathology dementia (Zotin et al., 2021). Although 2-VO is widely used in both VCI and ischemic stroke research, successfully modeling several aspects of human pathophysiology, its limitations must be acknowledged to guide future studies.

#### 2.1.2 Four-vessel occlusion with reperfusion

**Figure 3:**
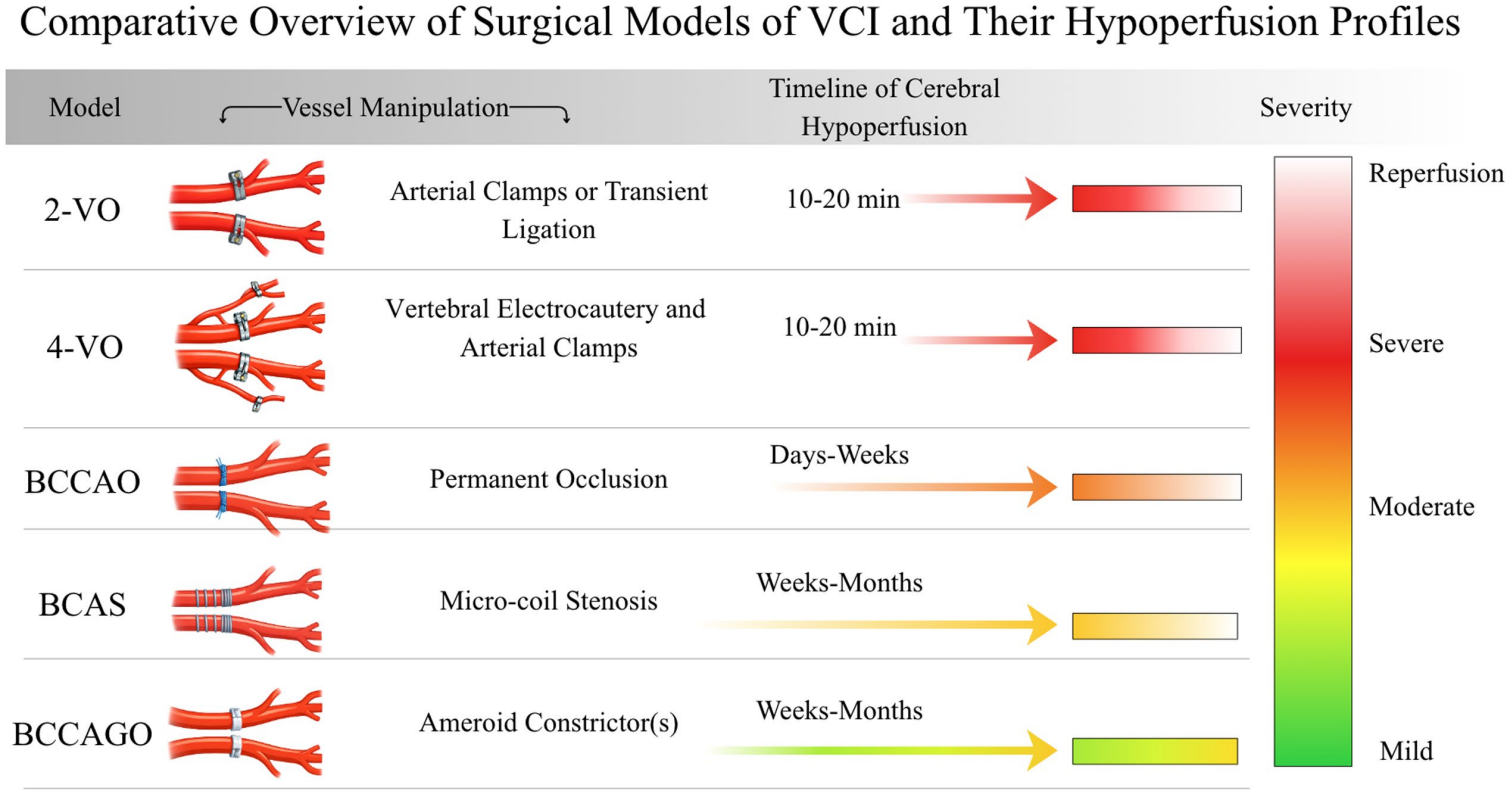
Comparative overview of commonly used surgical models of vascular cognitive impairment (VCI) and their respective hypoperfusion profiles. Models are organized by vascular manipulation procedure, duration of cerebral blood flow (CBF) reduction, and relative hypoperfusion severity. The two-vessel (2-VO) and four-vessel (4-VO) models produce transient global ischemia through temporary carotid clamping, with 4-VO also incorporating vertebral artery occlusion to generate more severe ischemia. Bilateral common carotid artery occlusion (BCCAO) induces chronic cerebral hypoperfusion (CCH) through permanent artery ligation. Bilateral common carotid artery stenosis (BCAS) produces CCH with lessened severity, applying micro-coils to the carotid arteries which stenose, rather than occlude, the vessel. Bilateral common carotid artery gradual occlusion (BCCAGO) utilizes ameroid constrictors to generate progressive arterial stenosis or occlusion. Arrows indicate approximate hypoperfusion timeline and the color scale indicates relative hypoperfusion severity across models.

The four-vessel occlusion (4-VO) model reduces CBF greatly and produces severe secondary hypoxia. This model was first described by Pulsinelli and Brierley (1979) with the goal of creating a model to override the rat’s well-adapted Circle of Willis. In 4-VO transient global ischemia models, both common carotid arteries are isolated through a ventral midline incision and are surgically clamped for 5–20 min using specialized atraumatic arterial clamps or hydraulic pressure occluders (Figure 3) (Pulsinelli and Brierley, 1979; Petito et al., 1998; Goto et al., 1990; Iwasaki et al., 1996). A second incision behind the occipital bone allows access to the bilateral vertebral arteries, which are permanently occluded (Pulsinelli and Brierley, 1979; Chung et al., 2002; Irie et al., 2002; Petito et al., 1998; Goto et al., 1990). Different from the 2-VO model, 4-VO results in a mixed model of permanent and transient occlusion with reperfusion. The 4-VO model indicates that histological changes and a marked reduction in cognitive integrity are relative to ischemic duration, highlighting a vital relationship between cerebral hypoperfusion and cognitive impairment via cell death (Chung et al., 2002; Irie et al., 2002; Neto et al., 2005; Pereira et al., 2012; Kim et al., 2023). These findings may be attributed to the occlusion of posterior vessels (i.e., the vertebral arteries) in addition to the forebrain ischemia which immediately and drastically reduces CBF to 12–14% of baseline during occlusion (Yamaguchi et al., 2005). Schmidt-Kaster et al., for example, evaluated regional CBF across numerous cerebral structures, noting reductions as severe as 2% of sham values in regions of the striatum and 6% of sham in the dorsal hippocampus when measured at 29 min (Schmidt-Kastner et al., 1989). Abrupt hippocampal CBF reductions caused by vertebral artery occlusion in 4-VO recapitulates features of hippocampal vulnerability consistent with strategic infarct-associated cognitive decline which result from large-artery disease of the vertebrobasilar system in patients of multi-infarct dementia (MID) (Kumral et al., 2015).

Similarly to 2-VO, 4-VO causes rapid and detrimental histological outcomes. 4-VO generates acute CA1 hippocampal neuronal apoptosis with WM degradation following 10-min transient common carotid occlusion and vertebral artery cauterization (Petito et al., 1998; Goto et al., 1990; Song et al., 2019). Neuronal survivability is negatively correlated with both the duration of vessel occlusion and the frequency of intermittent transient occlusion intervals (Chung et al., 2002; Pereira et al., 2012). A single 10-min ischemic interval produced no significant CA1 neuronal density reduction at 24 h post-reperfusion and a 45% reduction at 7 days. CA1 hippocampal neurons were reduced, however, by 75 and 79% when undergoing two and three 10-min ischemic intervals, respectively (Chung et al., 2002). In a later study by Song et al., CA1 hippocampal neuronal density declined to 106.4 ± 9.4 cells/mm^2^ as compared to 335.8 ± 10.0 cells/mm^2^ in the sham group, or approximately a 31% decrease in cell density (Song et al., 2019). Proportionally to the greater degree of ischemia, 4-VO reduces viable hippocampal neurons more drastically than is reported in both 2-VO and clinical dementia pathology. Acutely following reperfusion, the 2-VO model demonstrated insignificant hippocampal neuron loss; however, a 40% neuron loss was significant in the CA1, CA3, and dentate gyrus regions of the hippocampus in the 4-VO model (Wahul et al., 2018). CA1 neuron loss is correlated with poor composite memory scores (*r* = 0.62) in clinical dementia data where CA1 neuron populations are reduced by 48% as compared to non-Alzheimer’s patients (Zarow et al., 2005). The increased ischemic severity in the 4-VO, as compared to the 2-VO model, proportionally increases neuronal damage and, importantly, drives neuronal loss to clinical levels, which may indicate the value of 4-VO as a model to study the specific consequences of neuronal loss in relationship with dementia symptoms, specifically, key features of MID pathology, enabling investigation of ischemic dose-dependent neuronal injury with repetitive insult and mechanisms underlying MID and severe post-ischemic cognitive decline (Cummings, 1987).

As a primary protective cell for neurons, oligodendrocytes are also highly sensitive to ischemia, dying rapidly in the cortex and thalamus following ischemic injury (Petito et al., 1998; Walker and Rosenberg, 2010; Zhou et al., 2025; Pantoni et al., 1996). Healthy oligodendrocyte population reductions may contribute to secondary demyelination, resulting in poor synaptic integrity and progressive cognitive decline, as is observed in clinical vascular dementia pathology (Bouhrara et al., 2018; Jiang et al., 2017; Li et al., 2020). Oligodendrocytes were both reduced in number and morphologically abnormal by 48 h of reperfusion in ischemic stroke rodent models, consistent with 4-VO patterning (McIver et al., 2010). Increased MMP-2 and MMP-9 activity following both transient 2-VO and 4-VO ischemia suggests an important relationship between enzymatic BBB degradation and oligodendrocyte-associated demyelination that may be informative in the development of therapeutic targets (Lee et al., 2004; Walker and Rosenberg, 2010; Gorter and Baron, 2020). The adaptation of oligodendrocyte-targeting therapeutics in a 4-VO model of ischemia may offer insight into degenerative disease prevention following acute ischemic events such as cerebral infarction or multi-stroke dementia.

Abnormal neurometabolism is commonly identified in neurodegenerative diseases like AD and MID, and 4-VO ischemic injury recapitulates this disease. Schmidt-Kastner et al. reported increased free glucose concentrations in all evaluated brain areas at all circulation timepoints, indicating decreased glucose uptake or impaired metabolic activity as a consequence of hypoperfusion (Schmidt-Kastner et al., 1989). The authors found a near complete loss of ATP bioluminescence in the rodent forebrain acutely, with striatal ATP levels diminishing 24 h after reperfusion (Schmidt-Kastner et al., 1989). Glucose metabolites not only support ATP production as the primary energy source for the brain, but they also modulate various functions of neurons and glial cells. In 4-VO models, ATP depletion and glucose metabolite dysregulation can result in microglia activation, ROS production, and mitochondrial dysfunction which all contribute to dementia-like pathophysiology (Zhang et al., 2021).

In addition to neurometabolism dysfunction, neurotransmitter concentration is significantly reduced after reperfusion in 4-VO models, indicative of excitotoxic synaptic damage and compromised neuronal signaling (Iwasaki et al., 1996). In a study by Chung et al., acetylcholine activity was transiently increased during ischemia, but remained significantly decreased after 7 days, specifically in the frontal cortex and dorsal hippocampus (Chung et al., 2002). Conversely, dorsal hippocampus glutamate concentrations reached significant elevation by 7 days post-reperfusion, indicating metabolic imbalance with impaired reuptake (Iwasaki et al., 1996). Acetylcholine concentrations in cerebrospinal fluid are significantly lower in multiple-infarct dementia patients versus healthy controls, which was positively correlated with worse dementia scale scores (Tohgi et al., 1996). Evaluation of metabolite concentrations may serve as an early VCI marker, and the restitution of normal neurometabolism may offer a possible therapeutic target for future studies to evaluate (Tohgi et al., 1996). 4-VO induced neurotransmitter dysfunction exhibits similar patterns to 2-VO, supporting a pertinent role of neurotransmitter dysregulation in the development of working memory impairment seen in both models (Iwasaki et al., 1996; Wang et al., 2024; Lai et al., 2014).

Spatial working memory impairment, evaluated via the 8-arm radial maze, increased with ischemic duration following repeated occlusions under anesthesia, with deficits emerging early (within 24 h) and persisting 7 days following surgery (Figure 2) (Chung et al., 2002). Rats subjected to 10- and 20-min ischemic intervals, but not 5-min intervals, exhibited reduced spatial cognitive performance without significant impairment in mobility, suggesting that CCH-induced deficits in memory and cognition are dependent on ischemia duration (Iwasaki et al., 1996). As such, the degree of cognitive impairment appears proportional to the repetition of or total time under ischemia, with rats undergoing two 10-min occlusion intervals exhibiting greater reductions in 8-arm radial maze performance than rats subjected to one 10-min occlusion interval (Irie et al., 2002; Yamaguchi et al., 2005). Repetitive ischemic injuries resulting in cognitive deficits do modulate aspects of multi-infarct dementia, in which patients exhibit lowered working memory abilities and compromised executive functioning (Al-Adawi et al., 2014). Compounding ischemic intervals of 4-VO which correlate with more rapid or severe cognitive decline successfully recapitulate prominent outcomes of multi-infarct dementia, a leading contributor to VCI pathophysiology (Perez et al., 1975; Pereira et al., 2012).

##### 2.1.2.1 4-VO model considerations

Like the 2-VO transient global ischemia model, the 4-VO model employs temporary ischemia to evaluate the acute neural injury response. However, 4-VO intensifies cerebral hypoperfusion and subsequent damage with additional permanent vessel occlusion. Both models elucidate the brain’s acute response to hypoperfusion yet fail to recapitulate the progression of insidious disease pathology present within the VCI disease spectrum. The vertebral arteries are crucial in supplying posterior circulation to the rodent brain, such that ligation or occlusion of these vessels in addition to the carotid arteries generates severe ischemic injury across multiple brain regions, which synergistically contribute to poor cognitive outcomes (Lee, 1995; Aggarwal et al., 2012). Clinical studies have demonstrated marked reductions in working memory, perceptual processing, and global cognition, with infarct number and anatomical location representing critical determining factors in cognitive outcomes of MID (Aggarwal et al., 2012). Specifically, a study by Aggarwal et al. noted a 6-fold increase in dementia likelihood in patients with multiple infarcts across multiple regions (Aggarwal et al., 2012). Perhaps more importantly in positioning 4-VO as MID model, Aggarwal et al. suggest the deleterious effect of cortical, brainstem, and/or cerebellar infarcts in compounding dementia symptomatology, which are regions supplied most directly by the vertebral arteries in the rodent (Aggarwal et al., 2012; Lee, 1995).

4-VO studies primarily substantiate drastic CBF reductions, global neuroinflammation, metabolic disruption, and cognitive decline (Figure 3) (Iwasaki et al., 1996; Song et al., 2019; Schmidt-Kastner et al., 1989; Pulsinelli and Brierley, 1979; Petito et al., 1998; Neto et al., 2005; Pereira et al., 2012; Kim et al., 2023; Chung et al., 2002; Yamaguchi et al., 2005). Interestingly, specific WM lesions are poorly characterized in 4-VO models, with literature primarily emphasizing neuronal population loss in memory-associated cerebral structures. These principal outcomes align with clinical VCI but more accurately recapitulate cerebral infarction and reperfusion injury marked by neuronal apoptosis, proliferative neuroinflammation, and free radical generation within the ischemic penumbra (Fifield and Vanderluit, 2020; Zhao Y. et al., 2022). Rodent model WM changes are primarily evaluated via histopathology, whereas clinically, magnetic resonance imaging (MRI) identifies WM lesions and cerebral volume discrepancies (Kalaria, 2016). Gregory et al. evaluated T2-weighted (T2W) MRI changes following 4-VO ischemic injury in the rat, but the authors note challenges with rodent MRI such as lower resolution, false negative findings, and difficulty isolating structural subfields which may limit model reproducibility (Gregory et al., 2001). In fact, neither MRI nor laser doppler or speckle imaging are well utilized in 4-VO VCI models, thus greatly reducing the translatability of a major vascular dementia diagnostic component. Established in 1982, 4-VO was largely characterized before routine MRI adoption and the optimization of rodent imaging protocols, a phenomenon that may explain the lack of model-specific WM lesion characterization. Additionally, the 4-VO model is further limited by its extreme neurologic and ataxic rates (77%), proportional to ischemic degree (Pulsinelli and Brierley, 1979). In one study, a 20-min ischemic injury produces a 27.3% perioperative mortality rate with only 50% of the cohort surviving the 14-day study (Iwasaki et al., 1996). The close proximity of the vertebral arteries and the spinal cord may make it more difficult to determine if neurological deficits and higher mortality rates are secondary to the severity of the 4-VO procedure itself or incidental involvement of the spinal cord (Sun et al., 2021).

### 2.2 Chronic global ischemia

#### 2.2.1 Bilateral common carotid artery occlusion

While transient ischemic models such as 2-VO and 4-VO enable acute-phase evaluation of cerebral ischemia, chronic models have been developed to recapitulate progressive VCI pathology. Bilateral common carotid artery occlusion (BCCAO) is a well-characterized surgical VCI animal model, generating robust histopathological and cognitive outcomes following CCH induction. BCCAO recapitulates central features of CCH-driven subcortical ischemic vascular dementia (SIVD), characterized by prominent CBF reductions, progressive WM degeneration, basal ganglia compromise, and delayed cognitive deficits: hallmarks of SIVD pathophysiology involving the disruption of frontal-subcortical neural circuits (Schuff et al., 2009; Farkas et al., 2007; Cao et al., 2016; Choi et al., 2016; Schmidt-Kastner et al., 2005).

Chronic global hypoperfusion in the BCCAO model is achieved using fine suture material to permanently ligate both common carotid arteries (Figure 3) (Choy et al., 2006; Sarti et al., 2002; Wakita et al., 1994; Schmidt-Kastner et al., 2005; Bennett et al., 1998). The rat is preferential for this model over the mouse due to its well-developed Circle of Willis cerebral vasculature which permits ischemia but inhibits severe cerebral infarction (Farkas et al., 2007). The mouse Circle of Willis is poorly adapted with a complete structure present in only 10% of mice and limited to specific strains (Schröder et al., 2020; León-Moreno et al., 2020). BCCAO consistently instigates rapid and drastic reductions in global CBF and perfusion in rats. Schmidt-Kastner et al. showed that hippocampal CBF fell to 0.75 ± 0.06 mL/g/min (75% of sham) and the temporal cortex CBF was reduced to 1.32 ± 0.17 mL/g/min (57% of sham) 7 days after occlusion (Schmidt-Kastner et al., 2001). Choy et al. evaluated CBF alterations using continuous arterial spin labeling (ASL) MRI, notating a decrease in CBF in the cortex, hippocampus, and thalamus (Choy et al., 2006). CBF in these regions, however, was restored to insignificant differences from sham 6 months later (Choy et al., 2006). This vascular compensation is a natural limitation of rat VCI models due to their innate hemodynamically adaptive Circle of Willis which allows compensatory blood flow through basilar communicating arteries. While compensatory anastomoses is noted in clinical vascular dementia data, the incomplete human Circle of Willis challenges vascular compensation for frontal lobe ischemia, whereas the complete rodent Circle of Willis more readily restores global CBF in response to ischemic injury or persistent hypoxia (Wei et al., 2021). As such, angiogenesis and collateral vascular remodeling may offer promising therapeutic targets.

GFAP is often used as an indicator of astrocyte immunoreactivity (Schmidt-Kastner et al., 2005). Prominent populations of GFAP+ astroglial cells in the cortex, corpus callosum, and internal capsule arise as early as 1 week and persists in the corpus callosum through 6 months post-operatively (Figure 2) (Schmidt-Kastner et al., 2005; Farkas et al., 2004a). GFAP+ cell density is furthermore disproportionately increased in the CA1, CA3, and dentate gyrus of the hippocampus relative to other evaluated brain regions (Choi et al., 2016; Choi et al., 2011). Astrocytic end-feet play a critical role in the neuron glucose-lactate shuttle and are crucial to neurometabolic integrity especially in the context of ischemia or cerebral hypoxia (Verkhratsky et al., 2010; Li J. et al., 2016). In a BCCAO-induced state of CCH, astrocyte remodeling contributes to downstream neuronal apoptosis, synaptic metabolic disarray, and secondary cognitive decline consistent with clinical disease (Verkhratsky et al., 2010; Rodríguez et al., 2009; Choo et al., 2014). A BCCAO study by Lee et al. noted an increased concentration of pro-inflammatory cytokines such as COX-2, IL-1, and IL-6 within the dorsal hippocampus specifically (Lee et al., 2013). An acute increase in COX-2+ neurons of the hippocampus following ischemic induction throughout the CA3 and dentate gyrus indicates a reactive inflammatory response to the ischemic event itself rather than the chronic state of hypoperfusion (Farkas et al., 2004b). COX-2-related prostaglandins may play a role in the inflammatory development of atherosclerosis, a risk factor associated with vascular dementia development (de Bortoli et al., 2005; Wendell et al., 2012). Furthermore, the hippocampal endothelial cells are more susceptible to degradation than other brain regions, which is critically important given the increased global proliferation of pro-inflammatory cytokines following BCCAO (Johnson, 2023; Perosa et al., 2020; Lee et al., 2013). Neuroinflammation in chronic hypoperfusion models like BCCAO can be studied longitudinally to evaluate how specific regions are affected and what down-stream effects chronic inflammation may have on histology, metabolism, and cognitive function in VCI.

As a central immune cell, microglia are largely responsible for the recruitment and release of pro-inflammatory cytokines in the brain (Smith et al., 2012). Microglia are recruited and rapidly upregulated within 20 min of BCCAO-induced ischemic injury (Ábrahám and Lázár, 2000; Farkas et al., 2004b; Wakita et al., 1994). Marked gliosis, and the subsequent development of a glial scar, topographically align with MRI regions of interest, particularly the striatum (Soria et al., 2013). Persistent microglial activation indicates ongoing, perhaps progressive, neuroinflammation in response to CCH. In one study, CD4+/CD8 + lymphocytes (a subset of T cells with enhanced cytokine production) were present within the optic tract, internal capsule, caudoputamen, anterior commissure, and corpus callosum from 1 h to 90 days following complete vessel ligation (Wakita et al., 1994). These findings align with elevated cerebrospinal fluid levels of both CD4+ and CD8+ cells in both mild VCI and AD patients, with CD8+ T-cells most strongly correlated with clinical neuropsychological deficits (Lueg et al., 2015). Using the BCCAO model, Iba-1 + microglial activation was most densely present in WM, specifically the corpus callosum, internal capsule, and hippocampus (Choi et al., 2011; Wakita et al., 1994). Additionally, WM vacuolization on Kluever-Barrera staining was increasingly present in the corpus callosum and optic tract 14 days following vessel occlusion (Cho et al., 2006). Numerous studies report optic tract degradation as a key BCCAO model limitation (Farkas et al., 2004a; Tukacs et al., 2020). Farkas et al. reported 50% astrocytic disintegration in the optic tract with an accompanying 10-fold increase in microglial density as well as myelin sheath irregularities (Farkas et al., 2004a). The optic tract susceptibility to ischemic damage is largely attributed to the rodent WM architecture with both the corpus callosum and optic tract containing much of the rodent’s more limited WM density and the common carotids serving as the optic tract’s main vascular supply (Badea et al., 2007). Using the BBCAO model provides an opportunity to study the complex, synergistic interactions of neuroinflammation and WM damage that is persistent in VCI.

The high incidence of basal ganglia WM hyperintensities in clinical vascular dementia neuroimaging is recapitulated in the BCCAO model, making it a reliable model to target the therapeutic window of ganglia WM loss and its consequential altered striatal MRI and histological abnormalities (Acharya et al., 2019; Soria et al., 2013; Plaschke et al., 2006). Gold et al. reported a negative correlation between thalamic and basal ganglia lacunes with Clinical Dementia Rating scores, with silent lacunes ranging from 11 to 24% (Gold et al., 2005). WM lesions develop secondary to ischemic injury which may involve acute ischemic infarction, chronic small vessel disease, or mixed pathology. Within 24 h of BCCAO-induced ischemia, T2W MRI reveals unilateral areas of focal and acute damage to the striatum with bilateral lesions by 10 days (Soria et al., 2013; Plaschke et al., 2006). These findings are consistent with altered T2W distribution in human vascular dementia pathology (Van Straaten et al., 2004; Heiss et al., 2016). Typical lesions in clinical VCI often occur within the subcortical frontal WM and basal ganglia, with strategic infarcts focused in watershed areas surrounding the anterior, middle, and posterior cerebral arteries (Alber et al., 2019; Debette and Markus, 2010; Acharya et al., 2019). Furthermore, fractional anisotropy (FA) values are lower in the frontal cortex, thalamus, optic tract, and hippocampus in BCCAO rats versus sham at 24 h, indicating disorganized fluid movement from myelin fiber degradation or vessel lesioning (Soria et al., 2013; Wang et al., 2020). Histologically significant cerebral tissue (glial scarring and profuse gliosis) correlated with foci of greater axial diffusivity and increased FA, particularly within the striatum by 12 weeks post-ischemic induction (Soria et al., 2013). Additionally, diffusion weighted imaging (DWI) MRI revealed elevated apparent diffusion coefficient (ADC) signaling in BCCAO rats 3 days following occlusion, indicating free fluid movement from WM lesions and/or BBB (Sood et al., 2009). Similarly, there was a positive association between intima-media thickness of the carotid arteries and the temporal ADC which is correlated with greater WM hyperintensity (Altamura et al., 2016). Consistent with findings from Sood et al. using the BCCAO model, poor patient performance in neuropsychological memory and abstract reasoning is also associated with higher ADC in clinical cases (Altamura et al., 2016; Sood et al., 2009). Use of the BCCAO model in organisms with complex, gyrencephalic WM architecture could be invaluable for studying disease progression and therapeutic intervention windows due to the similarities even noted within lissencephalic animals.

The hippocampus is a largely preferred region of interest when evaluating histological changes in BCCAO models due to its vulnerability to ischemia and its role in memory formation and retention (Farkas et al., 2007; Tukacs et al., 2020; Li X. et al., 2016; Wang et al., 2020). Most specifically, the CA1, CA3, and dentate gyrus regions are sensitive to ischemic injury with apoptotic and necrotic pyramidal neurons observed from 2 to 12 weeks following vessel ligation (Bennett et al., 1998; Wang et al., 2020; Soria et al., 2013). Hippocampal atrophy is considered an early diagnostic hallmark in AD and is implicated in subcortical vascular dementia, though to a lesser degree (Li X. et al., 2016; Van De Pol et al., 2011). It is, therefore, important to consider mixed-pathology dementia when evaluating hippocampal outcomes in VCI animal models (Van De Pol et al., 2011; Johnson, 2023). In addition to region-specific neuronal apoptosis, hippocampal cellular metabolism of glucose and other energy substrates at the synaptic level was compromised in a chronically hypoperfused brain (Tukacs et al., 2020). These findings expand upon the acute metabolic imbalances noted in both 2- and 4-VO models (Heim et al., 2000; Matute et al., 2007; Wang et al., 2024; Schmidt-Kastner et al., 1989). While glucose concentration was elevated at 24 h in the 4-VO model, notably persistent glucose elevations at 3 weeks in the BCCAO model indicates neurometabolic malfunction secondary to CCH and ischemia (Schmidt-Kastner et al., 1989; Tukacs et al., 2020). Wang et al. noted an increase in the quantity of silent synapses (post-synaptic AMPA-silent) alongside reduced dendritic spine density in the hippocampal CA1 region following BCCAO (Wang Z. et al., 2016). The accumulation of extracellular glutamate may precede the spontaneous generation of AMPA-silent synapses, leading to progressive cognitive decline (Wang Z. et al., 2016; Hanse et al., 2013). Additionally, Ni et al. reported significantly decreased circulating acetylcholine (by 14.9%) in the striatum 1 month following BCCAO (Ni et al., 1995). By 4 months, acetylcholine concentrations decreased by 24.9% in the striatum, 21.2% in the cortex, and 14.5% in the hypothalamus as compared to sham (Ni et al., 1995). Furthermore, circulating choline, an acetylcholine precursor, decreased within the striatum, cortex, hypothalamus, and hippocampus, indicating a high likelihood of broad cholinergic dysfunction rather than acute neurotransmitter depletion (Ni et al., 1995; Zhao et al., 2015). Notably, vasoactive peptides (such as endothelin-1, CGRP, and vasoactive intestinal peptide) operate at the neurovascular interface, working synchronously with cholinergic and glutamatergic neurotransmitters in a dysregulated hypoperfused state (Staines et al., 2008). Given progressive deficits in functional and cognitive function despite eventual restitution of CBF, further investigation of the neurotransmitter-neuropeptide interaction in CCH may elucidate critical mechanisms underlying VCI progression and a possible therapeutic target (Tambo et al., 2025).

BCCAO model functional testing, particularly at chronic timepoints, correlates histopathological changes with progressive cognitive decline (Sopala and Danysz, 2001). At 60–90 days post-operatively, non-spatial working memory impairment was indicated by poor performance in novel object recognition testing (Farkas et al., 2007; Tukacs et al., 2020; Choi et al., 2016). Choi et al. demonstrated a 52.6% preference for the novel object, indicating random object choice rather than memory recollection (Choi et al., 2016). While impaired memory is a symptom of broad VCI presentation, it is no longer considered a principal implication of cognitive symptomatology (Van Der Flier et al., 2018). Early behavioral or neuropsychological changes in SIVD patients generally present as impaired attention, executive function, and verbal fluency, implicating prefrontal and subcortical neural pathways (Roh and Lee, 2014). Moreover, latency in the Morris water maze test and increased error occurrence in the 8-arm radial maze indicated impairment of both spatial working and reference memory (Farkas et al., 2007; Bennett et al., 1998; Kimura et al., 2025; Sopala and Danysz, 2001). Increased errors in 8-arm radial maze were apparent by 24 h after occlusion and subjects remained impaired through 21 days (Plaschke et al., 2001). Poor performance in the Y-maze was also observed at 60- and 90-days following BCCAO procedure. Performance discrepancies were also perceived at 30 days, but without statistical significance, thus suggesting a delayed onset of spatial working memory impairment following chronic blood flow reductions (Tukacs et al., 2020). Bennett et al. specifically correlated TUNEL-positive CA1 pyramidal neurons (i.e., apoptotic and late-stage necrosis) with Morris water maze and 8-arm radial maze errors at 27 weeks (*r* = 0.66 and *r* = 0.86, respectively) (Bennett et al., 1998). Spatial memory impairment worsens with increased study duration, supporting the hypothesis that chronic CCH causes more severe cognitive deficits than acute (Farkas et al., 2007). Poor performance in the elevated T-maze 30 days following vessel occlusion implicates dysfunction of inhibitory avoidance memory in the BCCAO rat (de Bortoli et al., 2005). Interestingly, a study by Atucha et al. evaluated inhibitory avoidance discrimination using a foot shock prior to and following the administration of a noradrenergic stimulant finding enhanced memory following treatment (Atucha and Roozendaal, 2015). Given both the proliferation of glutamate following 2-VO occlusion and the glutamate-mediated amplification of noradrenergic signaling, the continued exploration of neurotransmitter-associated cognitive outcomes in animal VCI models may reveal novel treatment targets (Atucha and Roozendaal, 2015; Mather et al., 2016). Evaluation of reference and working memory 16 months following BCCAO yielded a significant deficit in both memory types (Sopala and Danysz, 2001). By 16 months, sham rats begin to display memory decline attributed to normal aging. The significantly poorer performance of BCCAO rats indicates a synergistic effect of hypoperfusion with aging to accelerate cognitive decline. Importantly, the basal ganglia is also involved in task-relevant memory utilization and emotional regulation, the exploration of which may be important in future studies to connect cognitive decline with basal ganglia-specific WM ischemic injury (Frank et al., 2001). Additionally, impaired performance in fear and anxiety-dependent tasks correlate with neuropsychological changes in clinical vascular dementia symptomatology (Farkas et al., 2007; Sultzer et al., 1993). The elevated T-maze in rodent models examines inhibitory avoidance behavior relative to innate anxiety habits (Graeff et al., 1998). Abnormalities in elevated T-maze performance were also noted in stroke-prone hypertensive rats in a study by Ueno et al., suggesting a possible connection between vascular degeneration and behavioral changes (Ueno et al., 2002). Clinical findings note higher Neurobehavioral Rating Scale scores in vascular dementia patients as compared to AD patients, with higher incidence of anxiety/depression and behavioral abnormalities (Sultzer et al., 1993). Late-term onset of memory impairment in animal models highlights the need for a CCH model which can recapitulate the progressive decline seen in clinical VCI to more thoroughly investigate disease progression and mechanistic pathology.

##### 2.2.1.1 BCCAO model considerations

**Figure 4:**
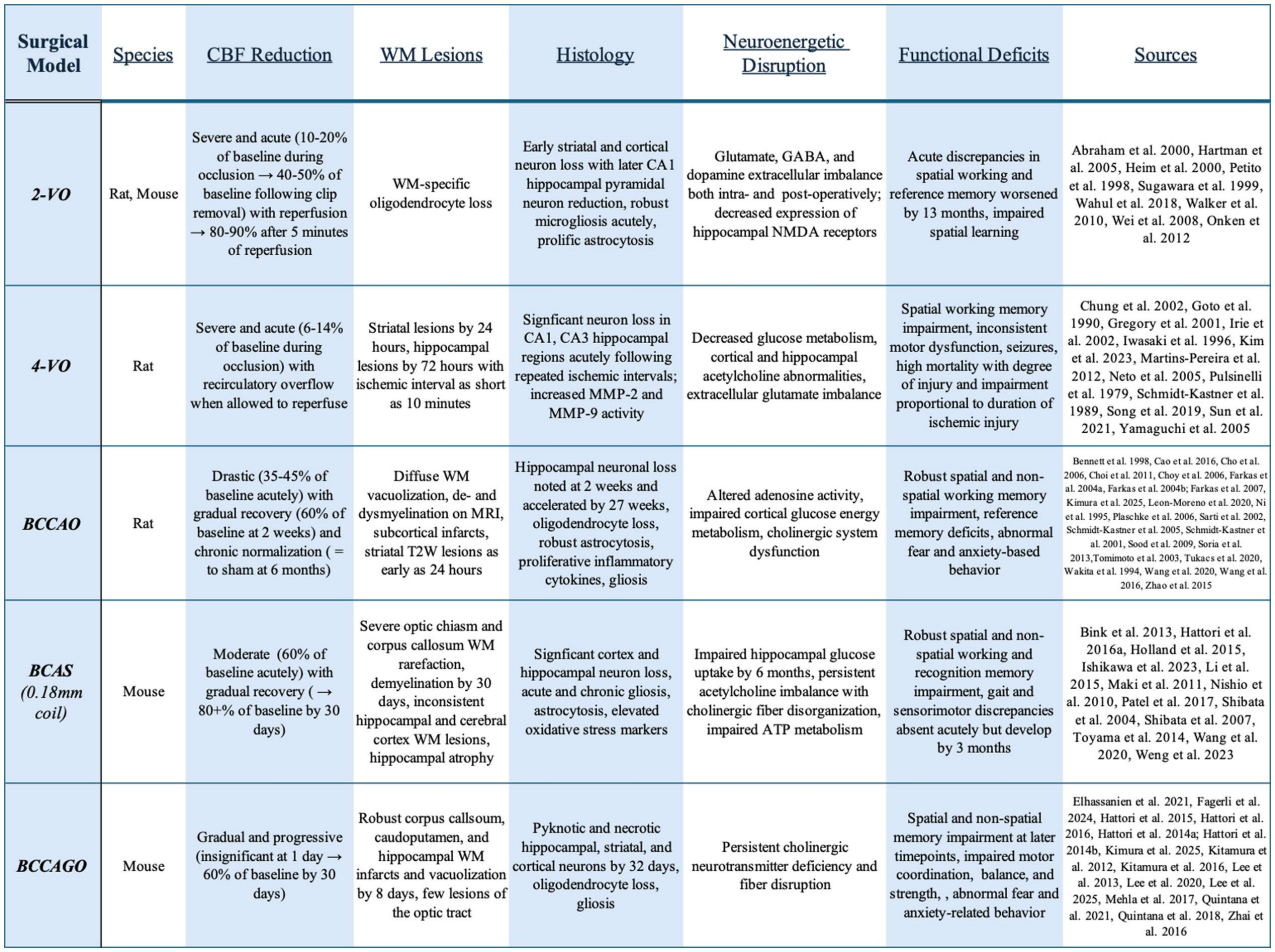
Summary of primary outcomes of surgical animal VCI models. Models include two-vessel occlusion (2-VO) and four-vessel occlusion (4-VO) transient global ischemia with reperfusion and bilateral common carotid artery occlusion (BCCAO), bilateral common carotid artery stenosis (BCAS), and bilateral common carotid artery gradual occlusion (BCCAGO) chronic global ischemia. Reductions in cerebral blood flow (CBF) vary from severe and temporary with reperfusion in 2- and 4-VO models to gradual and progressive in a BCCAGO model. White matter (WM) lesions, identified via histology, and WM hyperintensities, visualized via MRI, are reported for each model. Neurotransmitter and metabolic marker alterations are presented. Finally, cognitive, behavioral, and neuromuscular deficits are identified for each.

A complete evaluation of vascular dementia pathophysiology as it relates to outcomes noted in VCI animal models considers both acute injury response to ischemia as well as the chronic development of cellular and behavioral alterations (Figure 4). However, restitution of global CBF at chronic timepoints denotes a natural blood flow compensatory mechanism in rodents that poorly replicates human disease pathophysiology. Given the biologically advanced rodent Circle of Willis, CBF compensation likely occurs through dilation of basilar arteries and posterior communicating arteries (Choy et al., 2006). Significant differences in both length and tortuosity of the vertebrobasilar vascular system is present on magnetic resonance arteriography by 1 week and consistent through 12 weeks in BCCAO rats (Soria et al., 2013). Notably, changes in vessel tortuosity are observed in clinical SIVD. Emphasizing vessel integrity through longitudinal angiographic imaging in future investigations may clarify whether these vascular alterations in BCCAO models are a true feature of the disease or artifact introduced by surgical methods. This distinction may improve the reliability of the BCCAO model for recapitulation of SIVD pathology (Erkinjuntti, 2003; Román et al., 2002). Alterations in existent vasculature is furthermore accompanied by basilar angiogenesis (Soria et al., 2013). This vascular remodeling allows rodents to overcome some CCH symptomatology; however, it may indicate an interesting therapeutic target with further research surrounding compensatory angiogenesis.

A significant proportion of WM lesions occur in the optic tract to a greater degree than other brain regions of interest (Soria et al., 2013; Tukacs et al., 2020; Farkas et al., 2004a; Wakita et al., 1994). A loss of myelination fibers, vacuolization of WM tracts, and reductions in retinal neuron populations were evident in BCCAO rats after 12 weeks of occlusion (Soria et al., 2013). Due to the degradation severity in the optic tract, outcomes of functional testing, most of which require visual perception interpretation of cues, objects, and landmarks, must be considered in the context of compromised visual acuity. The 2-VO model, for example, has been applied to visual ischemic research context, such as diabetic retinopathy, but the continuation of the BCCAO model in VCI research requires some alteration to either preserve visual integrity or evaluate cognition without a dependence on visual cues (Farkas et al., 2007). Interestingly, however, visual impairment has been identified in AD pathophysiology with amyloid-β plaque deposition in the lens, nerve fiber degradation, axonal loss in the optic nerve, visual cortex pyramidal cell loss, and the presence of neurofibrillary tangles and tau pathology in the visual cortex (Marquié et al., 2019; Ferguson et al., 2024). Reduced visual function most certainly correlates with impaired cognitive and behavioral outcomes and quality of life in vascular dementia and AD patients (Zheng et al., 2024).

#### 2.2.2 Bilateral common carotid artery stenosis

While the BCCAO model of carotid ischemia is well tolerated in rats, the procedure is rather severe in mice and the survival rate is significantly reduced (Bink et al., 2013). Capitalizing on the poorly developed mouse Circle of Willis allows greater translatability to human disease, necessitating the adaptation of the BCCAO procedure to induce stenosis rather than total occlusion. Bilateral common carotid artery stenosis (BCAS) follows the same principal procedure as BCCAO in approaching the common carotid arteries via ventral incision, after which the vessels are separated from their sheaths and surrounding tissue. Rather than suture ligation, specialized micro-coils are twined around the exposed carotid arteries, just proximally to the carotid bifurcation, narrowing vessel luminal diameter to generate sustained stenosis and secondary cerebral blood flow reductions (Figure 3).

The micro-coils are made of specialized piano-wire material available in varying internal diameters, including 0.16, 0.18, 0.20, and 0.22 mm. The 0.18 mm internal diameter micro-coils are preferred as they allow blood flow reduction with lower mortality rates than 0.16 mm coils or occlusion (Ihara and Tomimoto, 2011; Khan et al., 2015). Reductions in CBF are not adequately achieved in the BCAS model when using a 0.22 mm internal diameter micro-coil (Ihara and Tomimoto, 2011; Shibata et al., 2004). At 2 h post-operatively, the 0.16 mm internal diameter coil group, however, more severely restricted CBF to 51.4 ± 11.5% of baseline when evaluated with laser doppler flowmetry (Shibata et al., 2004). The acute and severe CBF restriction using the 0.16 mm coil in mice resulted in extreme mortality rates equivalent to those of the BCCAO model when applied to mice (Shibata et al., 2004; Iwasaki et al., 1996; Bink et al., 2013). Furthermore, disproportionately high mortality rates (up to 75%) have been noted in 0.16 mm groups primarily resultant from large and severe cerebral infarcts (Ihara and Tomimoto, 2011; Toyama et al., 2014). The surviving animals with 0.16 mm coils displayed neurologic deficits and akinesia upon regaining consciousness (Shibata et al., 2004). In addition to drastic CBF reductions, the 0.16 mm internal diameter coils generated ischemic and apoptotic neurons in the cerebral cortex, hippocampus, and basal ganglia with parietal cortex cerebral infarcts in 60% of the surviving 0.16 mm group mice (Shibata et al., 2004). The most effective coil size in the BCAS model, 0.18 mm, sufficiently reduced CBF and generated secondary pro-inflammatory pathways and downstream neuronal degeneration while normalizing mortality rates to 15% (Bink et al., 2013). At 2 h post-procedure, CBF was reduced in 0.18 mm coil group mice to ~60 ± 10% of pre-operative baseline across studies (Shibata et al., 2004; Ishikawa et al., 2023; Gooch and Wilcock, 2016; Nishio et al., 2010; Kimura et al., 2025). CBF recovery trended back toward baseline by 1 day (72.4 ± 17.3%), but remained depressed relative to baseline and failed to recover as rapidly as transient occlusion and BCCAO models (Shibata et al., 2007). CBF was gradually restored to ~80% of baseline by 30 days (Ishikawa et al., 2023; Shibata et al., 2007) and to 85 ± 8.7% by 60 days (Nishio et al., 2010). Given the poorly developed mouse Circle of Willis, CBF restoration is hypothesized to recover via collateral angiogenesis or cerebral anastomosis (Ihara and Tomimoto, 2011).

CBF measurements obtained via arterial spin labeling (ASL) MRI rather than laser doppler flowmetry enable perfusion estimates of subcortical areas in addition to the cortical parenchyma. ASL revealed a more drastic and chronic CBF reduction to 50% of baseline at 14 days following BCAS with persistent reductions in thalamic and corpus callosum CBF at 50–60% of baseline at 3 months post-operatively (Ishikawa et al., 2023; Hattori et al., 2016a). Radial diffusivity (RD) was significantly increased in the hippocampus, corpus callosum, internal capsule, and striatum after 6 weeks of stenosis, indicating progressive myelin loss (Weng et al., 2023). Similarly, FA was altered in the frontal, temporal, and parietal lobes and in periventricular WM (Zhang et al., 2022). Thus, the persistent and moderate reduction in subcortical CBF in BCAS models reliably recapitulates CCH as a hallmark of VCI pathophysiology. Specifically, systematic clinical analyses of clinical small vessel disease (SVD) have shown a significant relationship between reduced mean blood flow velocity through the internal carotid and middle cerebral arteries and higher incidence of WM hyperintensity burden (Stewart et al., 2021). By reducing CBF without inducing acute ischemic infarction, the BCAS hemodynamic profile closely parallels SVD pathology, in which gradual WM degeneration occurs secondary to CCH and in the absence of overt large-vessel stroke (Yagita, 2024). BCAS may therefore provide a relevant experimental platform for modeling early vascular pathology which may predispose to subsequent stroke or progress to clinical SIVD.

Despite more conservative CBF reductions by BCAS as compared to BCCAO models, reductions in viable neuron populations and evidence of pyknosis and apoptotic neuronal signaling pathways are still apparent. In identical cortical slices, shams retained 85–90% viable neurons while identical regions of BCAS mice showed significantly diminished populations with 40% viable cells (Khan et al., 2015). When evaluated 8 months later, BCAS produced pyknotic neurons throughout both the cerebral cortex and hippocampus with preferential hippocampal atrophy (Nishio et al., 2010; Li et al., 2015). Cholinergic fiber disorganization and reduction was also present, suggesting compromise in neurotransmitter system integrity and reduced overall synaptic function (Nishio et al., 2010). A study by Ni et al. demonstrated significantly reduced acetylcholine levels in the striatum at 1 month following vessel ligation and in the striatum, cortex, and hippocampus at 4 months (Ni et al., 1995). A later study by Tanaka et al. correlated choline acetyltransferase (ChAT) and acetylcholine receptor deficiencies with poor discrimination learning task performance after 6 weeks of CCH (Tanaka et al., 1996). Post-mortem evaluation of patients with AD, multi-infarct dementia, or a mixed pathology dementia also revealed significantly reduced ChAT activity in the temporal, frontal, and hippocampal regions (Waller et al., 1986). Acetylcholine is synthesized by both neurons themselves and non-neuronal cerebral cells, specifically astrocytes, which are also significantly dysregulated in BCAS and BCCAO VCI models (Maurer and Williams, 2017; Bink et al., 2013; Ishikawa et al., 2023; Maki et al., 2011). Furthermore, muscarinic acetylcholine receptors are present both pre- and post-synaptically and are involved in heterogenous brain functions (Picciotto et al., 2012). Interestingly, muscarinic acetylcholine receptors can reduce glutamate release from cortical and striatal synapses (Picciotto et al., 2012). As such, the BCAS model may serve as a reliable and reproducible model through which cholinesterase inhibitors or cholinergic agents may be tested for therapeutic efficacy (Román and Kalaria, 2006; Wang et al., 2009).

In addition to neuronal density reduction, myelin staining intensity was reduced in 0.18 mm coil groups by 30 days following BCAS, most notably within the corpus callosum, caudoputamen, internal capsule, and optic tract, with myelin fibers appearing disordered and accompanied by frequent WM vacuolization throughout stained histological slices (Shibata et al., 2004; Shibata et al., 2007). WM lesions were not histologically apparent until 14 days, at which point rarefaction was minor, but noted in the corpus callosum, striatum, and internal capsule (Kimura et al., 2025). Severe lesions were present by 30 days and indicate a progressive loss of WM integrity following CCH induction (Shibata et al., 2004). Additionally, WM lesions were specifically noted in the hippocampus and corpus callosum of BCAS mice at 28 days with frequent and severe vacuolization, axonal loss, and reductions in CA1 hippocampal volume (Khan et al., 2015). While no gray matter infarctions or hemorrhages were noted in 0.18, 0.20, or 0.22 mm groups, partial cortical lesions were present in 0.16 mm groups at 30 days (Shibata et al., 2004; Shibata et al., 2007). Additionally, foci of glial accumulation with neuronal apoptosis and CA1 neuronal density loss accompanied vascular lesions in 0.16 mm groups (Shibata et al., 2004). Microglial populations increased from 7 to 30 days across all coil sizes, particularly those labeled for MHC class II antigen, which is implicated in chronic neurodegeneration (Shibata et al., 2004; Schetters et al., 2018). GFAP+ astrocytes were slower to activate, becoming significantly increased from 14 to 30 days in the 0.18 mm groups with correlation to regions of high density WM loss (Shibata et al., 2004). While glial cell activation was acutely elevated, but regressed to mild intensity 3 days following reperfusion in 2-VO models, the CCH induced in BCAS models allows for gliosis to remain increased, thus more accurately recapitulating the chronic gliosis of VCI and vascular dementia pathology (Ábrahám and Lázár, 2000; Alavez-Rubio and Juarez-Cedillo, 2024).

As endothelial adhesion molecules involved in immune regulation, ICAM-1 and VCAM-1 play a crucial role in signaling positive feedback loops for microglial and astroglial recruitment to areas of vascular ischemic injury or hypoperfusion (Chaudhari et al., 2025). Increased gene expression of both ICAM-1 and VCAM-1 was persistent through 28 days following BCAS procedure, correlating with increases in both GFAP+ cell density and Iba-1 + intensity at corresponding timepoints (Khan et al., 2015). Increased adhesion protein expression contributes to BBB degradation and cyclical neuroinflammation (Walker and Rosenberg, 2010; Madigan et al., 2016; Sweeney et al., 2018). MBP was drastically reduced in the hippocampus and corpus callosum 28 days after BCAS, consistent with WM lesion foci and abnormal myelin staining (Khan et al., 2015; Shibata et al., 2004; Xu et al., 2022). Concomitant GFAP+ cells and astrocyte proliferation were consistent with human vascular dementia pathology in which MMP-2 and GFAP^+^ cells accumulated around vascular infarcts and diffusely throughout WM (Rosenberg et al., 2001; Huang et al., 2014). Serum MMP-9 was also found to be elevated in patients with cognitive impairment in contrast to 2-VO models in which greater concentrations of MMP-2 compared to MMP-9 were found at acute timepoints (Zhao J. et al., 2022; Walker and Rosenberg, 2010; Lakhan et al., 2013). NO and extracellular potassium are recognized as key mediators of neurovascular coupling, making them promising targets for therapeutic intervention. The BCAS model may offer a platform for the exploration of endothelial NO synthase or other reactive oxygen species (ROS) scavengers given the overlap in pathological outcomes between BCAS and clinical SVD (Yılmaz and Bayraktutan, 2025).

Importantly, the consistent proliferation of oxidative stress markers in the BCAS model highlights the role of oxidative stress and neuroinflammation in human VCI and vascular dementia disease pathology. While acute elevations in cytokines and gliosis indicate neural reactivity to ischemic injury, the persistence of such factors correlate with chronic and progressive oxidative stress in patients of vascular dementia and AD, in which oxidative stress marker concentration is proportionally related to disease progression (Ansari and Scheff, 2010). Nitric oxide (NO), for example, is a bimodal intercellular messenger that, when in excess, is neurotoxic and triggers mitochondrial damage and cellular apoptosis (Zhang et al., 2025). Three weeks following BCAS procedure, both AMP and ATP were persistently elevated 8-fold from baseline, indicating impaired mitochondrial function and neurometabolic dysregulation (Plaschke et al., 2001). As a neurotransmitter under CCH, post-synaptic NO triggers glutamate release and modulates synaptic plasticity and long-term potentiation (Yang et al., 2025). Similarly, persistently prolific ROS and associated enzymes contribute to an apoptotic neuronal signaling cascade and endothelial tight junction claudin-5 and occludin degradation (Du et al., 2017; Tian et al., 2022). In a study by Holland et al., claudin-5 levels were directly assessed via enzyme-linked immunosorbent assay at 1 and 6 months following BCAS (Holland et al., 2015). Claudin-5 levels did not significantly differ from sham at 1 month, but were significantly decreased by 6 months (Holland et al., 2015). Cytokines such as (TNF-α) bind directly to neurons, triggering apoptotic neuronal pathways. TNF-α is elevated in both clinical AD and vascular dementia pathology (Tisato et al., 2016). In BCAS models, TNF-α was increased from 1 day to 4 weeks post-operatively, the direct stimulation of which correlates to a reduction in claudin-5 and occludin in cerebral endothelial cell populations (Toyama et al., 2014). These tight junction proteins are degraded by deleterious, upregulated MMPs, which, in turn, triggers neurovascular uncoupling and BBB disruption (Yang et al., 2007; Asahi et al., 2001; Ohtsuki et al., 2007). The intraperitoneal injection of Evans blue dye at 1, 3, 7, and 42 days following BCAS procedure by Yang et al. demonstrated tight junction disruption which allowed infiltration into the brain parenchyma, with Evans blue extravasation at all evaluated timepoints (Rajeev et al., 2022; Yang et al., 2022). Additionally, neuropeptide dysregulation represents an additional mechanism through which microvascular injury perpetuates VCI disease progression (Tambo et al., 2025). Tambo et al. reported upregulation of vasoconstrictive signaling molecules following BCCAO-induced CCH (Tambo et al., 2025). Modeling the oxidative and inflammatory cascade following CCH is vital in identifying the key factors perpetuating the degenerative cycle which leads to downstream tissue death and subsequent cognitive abnormalities.

The perpetuation of neuroinflammatory cytokines, astrocytosis, microgliosis, and progressive WM loss synergistically contribute to cognitive decline. Deficits in reference and spatial working memory have been reproduced through BCAS models (Patel et al., 2017; Ihara and Tomimoto, 2011). Several studies have also demonstrated working memory impairments in the 8-arm radial and Y-maze with marked increases in committed errors at 30 days (Shibata et al., 2007; Nishio et al., 2010; Toyama et al., 2014; Maki et al., 2011). Some mice were even unable to complete the 8-arm radial maze within the allotted time (Patel et al., 2017). Yang et al. reported BCAS mice committed to 20% more initial entry errors in the 8-arm radial maze versus sham (Yang et al., 2022). Relative to short-term memory, Khan et al. later showed impairments in BCAS non-spatial working memory in novel object recognition testing which correlated with prefrontal cortex histological abnormalities at a comparative timepoint (Khan et al., 2015). Changes in neural activation in clinical VCI testing often depends on working memory demands, thus correlating cognitive findings of BCAS models with patient outcomes (Kochan et al., 2010; Kirova et al., 2015). While the deep cortical WM most prominently impacted by CCH primarily correlates with executive function and working memory, gait abnormalities and imbalance present in clinical vascular dementia pathology indicate injury to the motor fibers responsible for normal ambulation (Moretti et al., 2011). A common neuropsychological clinical finding is the inability to walk-and-talk, or perform a dual motor and linguistic task (Montero-Odasso et al., 2012). At 30 days following coil placement, Shibata et al. did not obtain significant differences between BCAS and sham groups in sensorimotor reflex tests, gaiting, nociception, or wire hang and rotarod tests for motor coordination (Shibata et al., 2007). Importantly, the cognitive decline noted among mice is not accompanied by sensorimotor or coordination deficits at acute timepoints but instead develops by 3 months following vessel stenosis with significant discrepancies present in beam testing and stance percent of the gait cycle (Figure 4) (Shibata et al., 2007; Nishio et al., 2010). The addition of motor and discriminatory dual tasks to the BCAS model may strengthen the relationship of CCH-induced pathology and functional outcomes (Hernandez et al., 2020). Gait abnormalities and sensorimotor deficits are considerable symptoms of vascular dementia and AD, however, and the inability of BCAS models to produce motor deficits currently limits translatability (Montero-Odasso et al., 2012).

##### 2.2.2.1 BCAS model considerations

The BCAS model adequately models moderate, sustained hypoperfusion without inducing acute ischemic infarction noted in more severe occlusion paradigms such as 2-VO, 4-VO, and BCCAO. Milder reductions in CBF without large-vessel stroke makes BCAS a reliable model with which to investigate pathological features of SVD which precede CCH-induced VCI subtypes. The BCAS mouse model adequately reduces CBF, generating a CCH state without the severe optic tract WM rarefaction and myelin degradation as seen following BCCAO (Shibata et al., 2004; Soria et al., 2013). With optic tract integrity largely maintained, the robust discrepancies in cognitive testing outcomes can be interpreted with greater confidence than 2-VO, 4-VO, or BCCAO models which compromise the visual pathway with greater severity. Additionally, the milder reduction in CBF following BCAS as compared to BCCAO models and slower onset of secondary neuroinflammatory and immune cascades more accurately recapitulates patient VCI disease progression or vascular dementia pathophysiology. However, it remains more severe when considered against the true chronicity of the disease. Furthermore, endogenous vascular repair mechanisms may contribute to eventual CBF restoration in BCAS models due to the young age (10–12 weeks old) of rodents typically enrolled (Ihara and Tomimoto, 2011; Li et al., 2015). Given the prevalence of age as a VCI risk factor, young animal models introduce confounding factors that may reduce BCAS model applicability and translatability.

While BCAS is best suited for the exploration of SVD mechanisms and chronic outcomes within a pathophysiology-guided model selection framework, integration of genetic modifications, such as CADASIL or SHRSP, may enhance model dynamism and versatility, capturing additional key features such as atherosclerosis, impaired CBF autoregulation, and other microvascular abnormalities. It must also be acknowledged that BCAS achieves CCH through extrinsic large-vessel stenosis rather than small vessel failure, suggesting that SVD pathology is attained through indirect downstream consequence rather than direct capillary or small vessel manipulation. WM degradation, neuroenergetic dysregulation, BBB damage, and neuroinflammation observed in BCAS are largely secondary to uniform, global hypoperfusion (Ter Telgte et al., 2018). Within clinical SVD and vascular dementias, however, these processes arise from localized microvascular failure and heterogenous perfusion deficits. Consequently, BCAS may therefore overstate the direct impact of global CBF reduction and underrepresent the contribution of vascular remodeling, endothelial aging, and capillary network dysfunction (Ter Telgte et al., 2018).

#### 2.2.3 Bilateral common carotid artery gradual occlusion

Bilateral common carotid artery gradual occlusion (BCCAGO) involves the application of a specialized ameroid constrictor (internal diameter 0.5–0.75 mm) to one or both common carotid arteries (Figure 3) (Kimura et al., 2025; Hattori et al., 2016b). The ameroid constrictor consists of a stainless-steel ring filled with a hygroscopic casein material designed to absorb the fluid of the vessel it contains and swell until the vessel reaches near or total occlusion up to 28 days following constrictor placement (Hattori et al., 2015; Hattori et al., 2016b). Some studies employ an asymmetrical approach, placing an ameroid constrictor around one common carotid artery and a micro-coil (0.18 mm) around the other (Quintana et al., 2018; Hattori et al., 2016b). Common carotid artery stenosis can also be achieved using larger-diameter ameroid constrictors (0.75 mm internal diameter) to reduce luminal diameter rather than completely occlude the common carotid artery (Fagerli et al., 2024).

The BCCAGO model generates a delayed-onset, progressively declining CCH-state, more closely recapitulating gradual insufficiency associated with aging and chronic VCI diseases (Mokhber et al., 2021). This distinct feature of BCCAGO positions the model as a paradigm of progressive cerebral hypoperfusion, wherein vascular compromise evolves progressively rather than following a discrete ischemic event. As such, BCCAGO provides a valuable platform for investigating the temporal dynamics of vascular dysfunction, which may elucidate mechanisms underlying early-stage or subclinical VCI pathology and inform therapeutic strategies.

The BCCAGO model eliminates the drastic post-operative acute phase of CBF reduction with CBF remaining at baseline immediately following the procedure and not declining until 1–3 days later (Quintana et al., 2018; Hattori et al., 2014a; Kimura et al., 2025). Unlike BCCAO and BCAS models, which exhibit both drastic acute CBF reductions and eventual restoration of CBF, BCCAGO models demonstrate a continuous reduction in CBF (Hattori et al., 2015; Hattori et al., 2016b; Hattori et al., 2014a; Quintana et al., 2018). BCAS, for example, demonstrated an acute CBF reduction to 62.9 ± 18.5% of baseline at 2 h with recovery to 81.7% ± 4.0% at 1 month, while BCCAGO CBF declined from 83 ± 5% at 1 day to 49 ± 3% of baseline at 33 days (Fagerli et al., 2024; Nishio et al., 2010). In an asymmetrical BCCAGO study by Hattori et al., CBF declined to 87.2% of baseline on the ipsilateral constrictor side at 1 day and continued to decrease to 73.4% of baseline by 28 days (Hattori et al., 2015). A BCCAGO model using bilateral ameroid constrictor placement also noted CBF preservation compared to baseline at 2 h, 1 day, and 3 days at 98.2 ± 0.1%, 87.5 ± 2.2%, and 85.1 ± 3.9%, respectively. Similar findings between bilateral and asymmetrical ameroid constrictor placement may suggest hemispheric symmetry is not crucial to the development of global ischemic injury.

CBF reductions in BCCAGO models are proportional to both the internal diameter of the constrictor and the age of the animal. Notably, the placement of 0.5 mm ameroid constrictors around both common carotid arteries resulted in elevated mortality rates in mouse models (58.8% by 28 days) (Hattori et al., 2014a). In 1-year old mice undergoing bilateral 0.5 mm constrictor placement, only 20% of mice survive beyond 15 days (Hattori et al., 2014b). Additionally, CBF declined far more drastically in 1-year old cohorts versus 10-12-week-old animals (Hattori et al., 2014b). Because VCI risk increases proportionally with age, exaggerated CBF responses to carotid stenosis in older rodent models further support age as a key contributor to its pathophysiology (McVeigh and Passmore, 2006). Importantly, this gradual CBF decline more closely parallels cumulative vascular dysfunction rather than acute occlusion, which drives sustained injury and functional decline (Mokhber et al., 2021).

CBF is progressively reduced at both a cortical and subcortical level at 14–28 days when evaluated via ASL MRI in BCCAGO models (Hattori et al., 2015). Similarly, clinical evaluation of CBF using ASL demonstrated a reduction in thalamic, temporal lobar, and hippocampal CBF with correlations to WM hyperintensities and neuropsychological outcomes in patients of small vessel ischemic disease and mild cognitive impairment (Kalantari et al., 2025). At 28 days post-surgery, bilateral ameroid constrictor groups demonstrated global CBF rates of 70 ± 5% (Hattori et al., 2016b; Hattori et al., 2014a). But, subcortical WM appeared more sensitive to chronic ischemic injury with subcortical and cortical CBF at the bregma level reduced to 17 and 24% of baseline at 28 days, respectively (Hattori et al., 2015). Subcortical WM behaves similarly in acute post-stroke pathology, in which WM lesions predominantly occurred in deeper subcortical WM tracts (Hattori et al., 2015; Wang Y. et al., 2016). After 8 days of gradual occlusion, WM hyperintensities were apparent within the corpus callosum, caudate putamen, internal capsule, and hippocampal fimbria on DWI and T2W sequences (Hattori et al., 2015). WM hyperintensities were correlated with topographically equivalent subcortical infarcts, with most infarcts occurring within the caudoputamen and corpus callosum (Hattori et al., 2014a). Xie et al. noted statistically decreased FA and radial diffusivity (RD) in anterior subcortical regions of the aging human brain, indicating naturally progressive demyelination with an anterior–posterior gradient of WM deterioration in the aging population (Xie et al., 2016). The preferential susceptibility of subcortical WM to gradual hypoperfusion in BCCAGO models reflects patterns observed in both age-related cerebrovascular disease and healthy populations, where deep WM tracts (particularly anterior) demonstrated heightened sensitivity to sustained CBF reductions (Engelhardt et al., 2009; Xie et al., 2016). The presence of WM hyperintensities and subcortical lesions in older adult populations supports the relevance of the BCCAGO model to progressive, CCH-driven cerebral tissue degeneration (Wharton et al., 2015). Given the absence of MRI evaluation in the majority of VCI animal models, the significance of these subcortical lesions is difficult to attribute to the BCCAGO surgical model itself as they may be present in other models in which CBF evaluation is limited to cortical tissue. As the existing literature is presently limited, further characterization of BCCAGO multimodal imaging endpoints may strengthen its utility in evaluating chronic VCI disease in future studies.

BCCAGO models produce a comparatively lower level of gray matter damage, and subsequent metabolic changes, than other models such as BCCAO and BCAS (Kimura et al., 2025). Cerebral infarcts were identified via T2W MRI in numerous studies, particularly within the corpus callosum, caudate putamen, internal capsule, and hippocampus from 8 to 32 days post-surgery (Hattori et al., 2015; Lee et al., 2020). Hippocampal neurons appeared pyknotic and necrotic at 32 days ipsilateral to the ameroid constrictor, particularly within the CA1 and CA2 regions (Lee et al., 2020). Distinct from the CA1 and CA3 regions, which are selectively injured in BCCAO and BCAS chronic models, the CA2 region of the hippocampus is involved in social recognition memory and may be significant in the context of BCCAGO model behavioral and cognitive changes (Piskorowski and Chevaleyre, 2023; Bennett et al., 1998; Soria et al., 2013; Khan et al., 2015). Furthermore, the integrity of the optic tract is largely preserved in BCCAGO models with far fewer WM lesions noted 28–32 days following constrictor placement when compared to BCCAO models, strengthening the argument that cognitive changes in this model stem from CCH rather than visual compromise (Hattori et al., 2016b; Mehla et al., 2017). At 28 days, degenerative neurons displayed greater fluorescence intensity in the cerebral cortex, dorsal striatum, and CA1 hippocampus post-BCCAGO versus sham (Mehla et al., 2017). Reductions in healthy neuron populations within these brain regions may suggest reduced synaptic function necessary to perform executive function tasks, maintain healthy processing speed, and sustain attention. With rising diagnoses of vascular-related neurodegenerative diseases beneath the VCI umbrella, such as cerebral small vessel disease, vascular dementia, Parkinson’s disease, and multiple sclerosis, the milder, progressive cerebral changes of the BCCAO model may provide a useful experimental framework (Bohnen and Albin, 2011; Hanse et al., 2013; Lassmann, 2014).

Neuroenergetic disruption in vascular dementia-specific VCI is the result of an unsteady energetic flow to the brain, resulting in neural activity and functional connectivity deficiencies. As previously stated, BCCAGO models exhibit distinct neuronal loss, but ChAT+ neurons, in particular, were significantly reduced in both the dorsal striatum and medial septum of BCCAGO groups compared to sham, suggesting chronic cholinergic neuron deficiency (Mehla et al., 2017; Fagerli et al., 2024). Persistent cholinergic deficiency is generally accepted as a pathophysiological contributor to vascular dementia with both Goffries and Perry et al. reporting reduced ChAT+ neurons in brain tissue of vascular dementia patients (Waller et al., 1986; Gottfries et al., 1994; Perry et al., 1977). Zhai et al. found reduced striatal nicotinic acetylcholine receptor density at 8 months post-BCCAGO; however, muscarinic Ach receptor density showed no significant differences between sham and constrictor groups (Zhai et al., 2016). It is known that muscarinic Ach receptor populations are largely preserved in AD while nicotinic Ach receptors are significantly and consistently reduced in cortical tissue of AD patients (Nordberg, 2001). The extent of nicotinic cholinergic disruption in vascular dementia, however, remains ambiguous, as some studies demonstrate subcortical nicotinic receptor depletion while others note discrepancies only in “mixed” and “pure” AD pathology (Colloby et al., 2011; Perry et al., 2005). In conjunction with BCCAO-induced AMPA-silent synapse development, however, impairment of nicotinic Ach receptors in BCCAGO may indicate a pertinent role of synapse failure and neurovascular uncoupling as a driving force behind cognitive decline and should be explored within this model (Lecordier et al., 2021; Chen et al., 2022). Sparse BCCAGO literature underscores the need to clarify neurotransmitter and neurometabolic roles in this model. Broader application of BCCAGO to assess CCH-driven neuroenergetic shifts may illuminate mechanisms of cognitive decline beyond neuronal apoptosis and acute ischemic WM injury.

The BCCAGO model is uniquely able to recapitulate WM lesions and associated-glial cell abnormalities due to the use of gradual occlusion techniques. At 32 days post-operatively, demyelination was observed throughout the corpus callosum, concomitant with GFAP+ cell proliferation, Iba-1+ gliosis, and glutathione S-transferase (GST-π+) oligodendrocyte loss (Hattori et al., 2016b; Lee et al., 2020; Zhai et al., 2016). Zhai et al. specifically noted decreased myelin stain intensity at 2 (0.82 ± 0.04 vs. 1.00 ± 0.06, respectively) and 6 months (0.74 ± 0.10 vs. 0.91 ± 0.07, respectively) when compared to shams (Zhai et al., 2016). Hippocampal NeuN+ cells decreased in only 25% of BCCAGO mice receiving a 0.75 mm internal diameter constrictor with a 0.18 mm micro-coil, suggesting only a moderate incidence of hippocampal degeneration (Hattori et al., 2016b). Additional studies demonstrate prominent WM vacuolization and hippocampal neuron loss within the CA1, CA3, and dentate gyrus of the hippocampus in 0.5 mm constrictor groups (Quintana et al., 2018). Discrepancies in histological results may be due to the internal diameter of the ameroid constrictor applied. Additionally, axonal injury and prominent corpus callosum atrophy are notable in 0.5 mm ameroid constrictor models (Quintana et al., 2018). Axonal injury in VCI pathology is generally attributed to Wallerian degeneration of cortical pyramidal neurons to the corpus callosum (Tomimoto et al., 2004; Yamauchi et al., 1993). Tomimoto et al. noted prominent corpus callosum atrophy without histological lesions in AD patients, but emphasized only mild corpus callosum atrophy with deep WM lesions in patients with small and large vessel ischemic disease (Tomimoto et al., 2004). Severe body atrophy and deep WM lesions in BCCAGO models adequately recapitulate those seen in clinical patients, supporting their efficacy as a model of mixed-pathology dementia.

BCCAGO models aim to correlate CBF reductions and histological damage with overt and progressive cognitive impairment, particularly proportional with the severity of disease. Spatial working memory deficits were apparent at 28 days following ameroid constrictor placement, evidenced by discrepancies in exploratory alternation between Y-maze arms (Kimura et al., 2025; Hattori et al., 2015; Hattori et al., 2016b; Hattori et al., 2014b). Consistent with low incidence of hippocampal neuron loss, equivalent performance in the Morris water maze between sham and BCCAGO groups indicated retention of hippocampal-dependent reference memory at the same timepoint (Hattori et al., 2016b). Meanwhile, Fagerli et al. reported no significant Y-maze discrepancies or neuronal population changes between sham and BCCAGO mice at 33 days post-procedure using 0.75 mm internal diameter constrictors (Fagerli et al., 2024). In future studies, greater diameter constrictors may require longer survivability timepoints to achieve clinically comparable histological and cognitive outcomes. Despite the lack of statistical significance, some obvious differences in Morris water maze performance between sham and 28-day post-BCCAGO groups, such as longer escape latency and reduced swimming speed, indicated a reduction in typical motivation behavior (Hattori et al., 2014b). Interestingly, Hattori et al. reported reduced swimming speed in ameroid constrictor groups versus sham, which is not a common finding in other VCI models despite robust discrepancies in performance, path length, and time in target area (Hattori et al., 2014a; Ma et al., 2023; Holland et al., 2015). BCCAGO mice exhibited reduced freezing in delayed tone fear conditioning, reflecting impaired anxiety-related behavior and aligning with the heightened impulsivity observed in vascular dementia patients (Mehla et al., 2017; Ueno et al., 2002; Sakurai et al., 2020). Furthermore, poor performance in novel object recognition indicated that CCH impaired both recognition and declarative memory as well as innate exploratory behavior (Mehla et al., 2017). Novel object recognition testing may also be used to challenge and evaluate short- and long-term memory, as both forms of memory tasks rely on the medial temporal lobe (Kumaran, 2008; Sarhan et al., 2025). Discrepancies in the novel object test not only in BCCAGO groups, but also across other surgical VCI models, indicated strong positive correlations between temporal lobe (e.g., frontal cortex and hippocampus) injury and both acute and progressive cognitive decline (Fagerli et al., 2024; Farkas et al., 2007; Choi et al., 2016; Khan et al., 2015; Tukacs et al., 2020).

Interestingly, motor coordination, balance, and muscle strength were all implicated in BCCAGO mice at 28 days as indicated by both shortened latency to fall in rotarod and wire hang tests (Figure 2) (Hattori et al., 2016b; Hattori et al., 2014b; Zhai et al., 2016). In a clinical cohort study by Duchowny et al., reduced grip strength was associated with poor memory test performance, volume of WM hyperintensity, and a vascular dementia diagnosis (Duchowny et al., 2022). Hattori et al. recapitulated the neuromuscular weakness associated with VCI pathology by reporting significantly shortened latency to fall in the wire hang test at 28 days (Hattori et al., 2014a). Furthermore, Mehla et al. demonstrated longer crossing times on the balance beam with greater incidence of foot slips at the same timepoint, thus suggesting decreased motor coordination and balance secondary to constrictor-induced CCH (Mehla et al., 2017). Motor coordination is associated with human vascular dementia pathophysiology in which lacunae and deep WM tract lesions in the frontal, parietal, and temporal cortices propagate reductions in executive function integrity, motor coordination, balance, and gait abnormalities (Kim et al., 2025; Elhassanien et al., 2021). Furthermore, a compromised basal ganglia may perpetuate both motor discrepancies and motivation or emotional regulation capacity (Kim et al., 2025). Although injury-driven cerebral compensation and reorganization complicate linking cognition and motor function, BCCAGO models may help clarify the anatomical cand pathological correlations of VCI spectrum diseases.

##### 2.2.3.1 BCCAGO model considerations

A recent comprehensive review by Santisteban and Iadecola attributed vascular aging to 7 main mechanisms: renin-angiotensin system upregulation, mitochondrial dysfunction, free radical proliferation, vascular senescence, extracellular matrix remodeling, persistent neuroinflammation, and depletion of stem cell reserves. Notably, many of these pathological processes have been observed and modeled in BCCAGO studies, highlighting the relevance of BCCAGO models in recapitulating the multifactorial nature of age-related vascular dysfunction. In capturing these converging mechanisms, BCCAGO serves as an experimental platform for investigating the complex interplay underling aging and CCH-mediated VCI disease (Santisteban and Iadecola, 2025).

BCCAGO-induced gradual CBF reduction, with no spontaneous restitution, more reliably recapitulates human small vessel disease while generating abundant secondary histological and immunohistochemical injury data (Figure 4). The milder histological findings in BCCAGO groups suggest a greater impact of chronic oligemia on neuroinflammation resultant from subcortical CBF reductions rather than acute cortical ischemia (Hattori et al., 2015; Kitamura et al., 2012). In mouse studies, the model is constrained by high mortality, since survival beyond full constrictor occlusion is limited by the poorly developed Circle of Willis, yielding mortality rates similar to those that preclude BCCAO models (Hattori et al., 2015; Bink et al., 2013; Hattori et al., 2016b; Lee et al., 2020). However, models employing ameroid constrictors thus far appear to effectively reduce the incidence of confounding visual factors while demonstrating etiological mechanisms and pathophysiological changes with translatable symptomatology to human VCI. Specific WM tracts are implicated in studies applying the BCCAGO model which correlate most robustly with gradual CBF reductions and consequential deficits in learning, memory, and cognition. Furthermore, ameroid constrictor models note the development of gait abnormalities and muscular weakness, which is not apparent in most BCCAO and BCAS models, reflecting key aspects of cerebrovascular aging which are not captured in other paradigms. These findings may facilitate earlier diagnosis as gait abnormalities are predictive of clinical vascular dementia (Beauchet et al., 2016). Gradual CBF reductions without compensatory restitution more reliably recapitulate CCH in human vascular dementia than BCCAO or BCAS models, which instead demonstrate acute drastic ischemic injury. While CCH model refinement has elucidated the heterogenous underlying mechanisms of progressive VCI disease relative to WM injury and neuroinflammatory cascades, most models still struggle to recapitulate gradual disease onset. The BCCAGO model most adequately produces gradual and permanent CCH and demonstrates relevant immunohistochemical consequences.

Despite strong translational potential, the BCCAGO model remains comparatively underrepresented in current VCI literature relative to more established paradigms such as BCCAO and BCAS. Existing studies utilizing constrictor-induced gradual occlusion techniques are few, largely restricted to rodent cohorts with relatively short experimental timelines, leaving longitudinal characterization of progressive WM degeneration, neurovascular disruption, neurotransmitter dysregulation, and associated cognitive and functional decline incomplete. The standardization of surgical procedure, ameroid constrictor dimensions, and post-operative imaging protocols is also lacking across existing studies, complicating direct cross-study comparability. However, the gradual hemodynamic decline and the avoidance of both abrupt ischemic infarction and severe perioperative mortality rates enables more precise investigation of chronic neurodegenerative mechanisms. Consequently, the limited literature surrounding BCCAGO should not be interpreted as a model weakness but rather as an important gap in the field and strong opportunity for continued model optimization, longitudinal characterization, and translation into large animal systems.

## 3 Limitations and translational barriers in VCI modeling

Given the limited WM density in lissencephalic rodent models, the necessity for large animal models with improved clinical translatability is increasingly apparent (Krafft et al., 2012; Zhou et al., 2025; Washida et al., 2019; Hietamies et al., 2018). Due to the size and simplicity of the rodent brain, deep WM lesions, and CBF are challenging to evaluate and do not fully recapitulate clinical pathology (Denic et al., 2011; Hietamies et al., 2018). Additionally, though the rodent model is economically attractive and easily replicated, the lissencephalic rodent brain lacks the intricate cortical folding and corresponding WM vulnerability characteristic of gyrencephalic brains, reducing translatability to clinical disease. The relative scarcity of large animal models is generally attributed to cost barriers, technical demands, and ethical considerations, resulting in the continued reliance on rodent systems which incompletely capture key aspects of VCI pathophysiology and progression (Ribitsch et al., 2020). The lissencephalic rodent brain limits CBF exploration as compared to the gyrencephalic brain due to its lack of gyri folds, significant decrease in WM concentration, and lack of deep WM. The areas of greatest WM density in the rodent brain are the corpus callosum, internal/external capsules, and optic chiasm, which function as the regions of interest most often evaluated for WM injury and degradation (Zhou et al., 2025). The limited evidence of gross WM changes sub-optimally recapitulates injury to deep cortical WM noted in human pathology (Yang et al., 2016), though many models effectively elicit WM rarefaction as a substantial building block for pathophysiological understanding. The restitution of CBF is largely attributed to species-specific cerebrovascular anatomy differences, particularly the Circle Willis, which is more complete and functional in rodents than in humans (Schröder et al., 2020). The variability in Circle of Willis vascular anatomy may underestimate long-term white matter injury in rodent models. Furthermore, the incomplete or variable anatomy of the human Circle of Willis contributes to sustained regional hypoperfusion, particularly frontal-subcortical, which drives chronic ischemic subtypes of VCI such as SIVD and SVD. Furthermore, most surgical VCI models induce CCH in otherwise young, healthy animals, whereas clinical VCI develops in aged patients with largely complex vascular and metabolic comorbidities including hypertension, diabetes, atherosclerosis, obesity, hypercholesteremia, and dietary and lifestyle factors (Mahinrad et al., 2023; Temedda et al., 2025). These comorbidities influence endothelial function, health of the neurovascular unit, collateral cerebral circulation, BBB integrity, and WM vulnerability. Isolated hypoperfusion models may thus underestimate both disease severity and heterogeneity which is observed clinically. These discrepancies between rodent models and clinical outcomes limit direct translation of experimental findings in preclinical models and substantiate the need for large animal models with comparable cerebrovascular architecture. Given the intricate molecular mechanisms underlying VCI development and progression, a multifaceted approach will be a necessary strategy in generating future translatable models development, preclinical and clinical trial optimization, and reliable novel biomarker identification for vascular cognitive disease.

In clinical evaluation, MRI is frequently used to establish baseline and pathologic deviations in CBF. Susceptibility-weighted imaging (SWI) identifies cortical and subcortical hemorrhage while T1-weighted imaging detects atrophy patterns. Fluid-attenuated inversion recovery imaging (FLAIR) is best utilized to demonstrate WM hyperintensities and identify deep periventricular or subcortical WM lesions (Raji and Benzinger, 2022). MRI is not an overtly practical radiological imaging approach in rodent models, as resolution issues and artifact frequency are common challenges due to small brain size (Denic et al., 2011). Rodent models primarily employ laser speckle imaging and laser doppler flowmetry to evaluate CBF but must rely more heavily on histology for WM changes. This is a major consideration as repeated measures cannot be performed on individual animals longitudinally over disease progression, thus preventing researchers from identifying potential diagnostic biomarkers. Functional MRI (fMRI) is a clinical MRI technique used to image blood flow and oxygen perfusion and is generally performed on conscious patients. However, due to the limitations of animal models, fMRI is difficult to perform in a directly translatable manner to clinical patients. Yet, modern use of large animal models has made it possible to evaluate resting-state fMRI changes in both healthy brains and injured brains *in vitro*, longitudinally (Ahmed et al., 2025; Simchick et al., 2021; Simchick et al., 2019). Furthermore, the small rodent brain size requires field strength of approximately 4.7–11.7 T versus the standard clinical 1.5-3 T, thus generating a higher incidence of image artifact and noise (Denic et al., 2011). Limited animal studies have demonstrated substantial subcortical CBF reductions using ASL, providing compelling evidence that MRI-based techniques offer a more reliable and precise representation of CCH-induced WM injury. The distinction in these WM populations must be made when considering the efficacy of CBF reduction in animal models, particularly those in which subcortical structures are challenging to image due to small brain size and low architectural complexity in rodents.

Current clinical assessment of VCI-associated pathophysiology in human medicine relies more heavily on the neuropsychological evaluation of executive function, attention, memory, language, and visuo-spatial function than imaging mechanisms, thus necessitating an animal model that can replicate functional and cognitive deficits and confirm histological correlations (Skrobot et al., 2018; Jiwa et al., 2010). When subjected to various executive skills tests, patients with vascular dementia are found to have profound reductions in executive function with a strong correlation between advanced neuropsychological symptoms and reduced daily independence (Chen et al., 1998). Functional outcomes are cornerstones to establishing and improving human quality of life following the onset of AD, vascular dementia, or post-ischemic small vessel disease. Establishing baseline functional outcomes from which subsequent treatment targets or prophylactic treatments can be developed will be critical for improving VCI prognosis.

Future VCI paradigms should more rigorously investigate sex as a biological variable influencing pathophysiological and cognitive outcomes, particularly given the disproportionate diagnosis incidence and clinical burden of AD in females (Wood Alexander et al., 2025; Akhter et al., 2021; Lin and Doraiswamy, 2015). Growing evidence supports sex-specific vascular, hormonal, metabolic, and inflammatory risk factors for cardiovascular and neurodegenerative disease and an increased susceptibility to VCI-adjacent comorbidities (Wood Alexander et al., 2025; Gannon et al., 2019; Liu et al., 2026). Existing clinical studies have identified sex-dependent discrepancies in cognitive performance, WM lesion burden, progression of functional decline, and neuropsychological presentation, particularly in mild VCI cases (Xing et al., 2012; Akhter et al., 2021; Lin and Doraiswamy, 2015). These dimorphisms are particularly important in aging populations, where peri- and post-menopausal estrogen depletion may influence mechanisms underlying VCI disease progression (Gregory et al., 2025; Mervosh and Devi, 2025; Nguyen et al., 2021).

Despite these clinically relevant indications, the majority of VCI surgical models utilize exclusively young male cohorts, limiting the translational application of preclinical findings to both sexes. Consequently, existing VCI preclinical data may incompletely characterize sex-dependent mechanistic contributions to disease development and progression. Future preclinical models should prioritize inclusion of both sexes to improve translatability and fidelity to the patient populations within clinical disease.

VCI animal models have been successful in recapitulating pathophysiology associated with clinical dementia at acute and chronic timepoints relative to study duration. Older rodents have been used in some studies, but baseline cognitive errors are typically elevated, making it challenging to extrapolate significant data (Schmidt-Kastner et al., 2005; Wang et al., 2020; Daneshjoo et al., 2022; De La Torre and Fortin, 1994). Additionally, the lack of chronic studies limits the evaluation of disease progression. Rodent VCI models consistently demonstrate a natural CBF return to baseline between acute and chronic (14–30 days) timepoints (Shibata et al., 2004; Jiwa et al., 2010; Washida et al., 2019; Ishikawa et al., 2023). Most models conclude 30 days post-procedure, at which point moderate CBF restoration is noted. Chronic models (>6 months) better replicate human pathophysiology by noting persistent CBF reductions and linearly related frontal cortex lesions, hippocampal atrophy, and cognitive deficits (Ihara and Tomimoto, 2011). Future studies should emphasize evaluation of chronic cerebral hypoperfusion to recapitulate long-term disease progression in clinical settings.

## 4 Conclusion

Advancing the translational relevance of future VCI research will require a shift from generalized model availability-driven study design toward intentional, pathophysiology-aligned model selection. The framework proposed in this review, utilizing available VCI models, offers a foundation for this transition. For example, integrating complementary model systems may offer insight into mechanisms underlying mixed dementias, which capture a substantial percentage of VCI-diagnosed individuals, and can be achieved by combining approaches. Pairing an acute ischemic paradigm with a chronic model may capitalize on the interplay between acute vascular injury and delayed WM degeneration observed clinically, strengthening the mechanistic understand of and therapeutic strategy generation for VCI subtypes such as PSD and MID. The expanded use of large animal models represents another critical future direction. With greater WM volume and complexity, gyrencephalic architecture, and comparable cerebrovascular organization, an established porcine or non-human primate model would improve accuracy and translatability of experimental findings.

Future work should also emphasize longitudinal studies using multimodal imaging approaches. Incorporating techniques such as ASL, diffusion tensor imaging (DTI), and fMRI may strengthen direct comparisons between animal and clinical disease outcomes, particularly WM integrity, connectivity, and CBF dynamics. Establishing shared imaging endpoints across preclinical and clinical studies may facilitate the identification of novel biomarkers to improve therapeutic efficacy.

The 2017 VICCCS reclassification of VCI into distinct subtypes has necessitated the classification of surgical animal models of VCI accordingly to enhance mechanistic understanding, optimize therapeutic strategies and interventions, and improve the clinical relevance and translatability of investigative findings (Skrobot et al., 2017). Rather than categorizing surgical animal VCI models as interchangeable representations of a broad disease spectrum, this review emphasizes the need to position each model within a pathophysiology-guided framework dependent on specific mechanisms of disease subtypes. Within this framework, transient ischemic models such as 2-VO are best suited to investigate acute ischemia, reperfusion injury, and the onset of PSD while 4-VO most closely replicates features of MID relative to cumulative ischemic burden. Chronic hypoperfusion models such as BCCAO more closely recapitulate numerous SIVD and SVD pathologies such as WM degeneration and delayed cognitive decline. The newer BCCAGO model is best suited for the investigation of gradual CBF reduction and vascular aging. These distinctions between models underscore a defining challenge of VCI research: no single model yet captures the full spectrum of disease mechanisms or outcomes; yet each provides specific insight into aspects of the heterogenous pathophysiology which has driven subtype delineation. As also emphasized in this review, treatments and prophylactic therapies have been difficult to develop due to the complex VCI heterogeneity and its deleterious progression from initial onset. With no perfect model to recapitulate human VCI, model selection has proven pivotal for desired disease outcomes and intended investigation. As the incidence of AD, VCI, and other spectral dementia pathologies continue to rise, there is a proportionally urgent need to investigate underlying mechanisms and pursue novel therapies, reliant on strategic model selection for more precise, hypothesis-driven investigation.

## References

[ref1] Ábrahám H. Lázár G. (2000). Early microglial reaction following mild forebrain ischemia induced by common carotid artery occlusion in rats. Brain Res. 862, 63–73. doi: 10.1016/S0006-8993(00)02072-2, 10799670

[ref2] Acharya A. Liang X. Tian W. Jiang C. Han Y. Yi L. (2019). White matter Hyperintensities relate to basal ganglia functional connectivity and memory performance in aMCI and SVMCI. Front. Neurosci. 13:1204. doi: 10.3389/fnins.2019.01204, 31798401 PMC6874172

[ref3] Aggarwal N. T. Schneider J. A. Wilson R. S. Beck T. L. Evans D. A. De Carli C. (2012). Characteristics of MR infarcts associated with dementia and cognitive function in the elderly. Neuroepidemiology 38, 41–47. doi: 10.1159/000334438, 22179433 PMC3254097

[ref4] Ahmed I. Reeves W. D. Laballe M. H. Taber M. F. Sneed S. E. Kaiser E. E. . (2025). A novel integration of brain structural and functional connectivity for identifying traumatic brain injury induced perturbations. J. Neurosci. Methods 419:110459. doi: 10.1016/j.jneumeth.2025.110459, 40273994 PMC12103726

[ref5] Akhter F. Persaud A. Zaokari Y. Zhao Z. Zhu D. (2021). Vascular dementia and underlying sex differences. Front. Aging Neurosci. 13:720715. doi: 10.3389/fnagi.2021.720715, 34566624 PMC8457333

[ref6] Al-Adawi S. Braidy N. Essa M. Al-Azri F. Hussain S. Al-Sibani N. . (2014). Cognitive profiles in patients with multi-infarct dementia: an Omani study. Dement. Geriatr. Cogn. Disord. Extra 4, 271–282. doi: 10.1159/000363621, 25202321 PMC4154192

[ref7] Alavez-Rubio J. S. Juarez-Cedillo T. (2024). Microglia as a possible alternative therapeutic for dementia. J. Alzheimer's Dis. Rep. 8, 43–56. doi: 10.3233/ADR-230112, 38229830 PMC10789290

[ref8] Alber J. Alladi S. Bae H.-J. Barton D. A. Beckett L. A. Bell J. M. . (2019). White matter hyperintensities in vascular contributions to cognitive impairment and dementia (VCID): knowledge gaps and opportunities. Alzheimer’s Dement. 5, 107–117. doi: 10.1016/j.trci.2019.02.001 PMC6461571 31011621

[ref9] Altamura C. Scrascia F. Quattrocchi C. C. Errante Y. Gangemi E. Curcio G. . (2016). Regional MRI diffusion, White-matter Hyperintensities, and cognitive function in Alzheimer's disease and vascular dementia. J. Clin. Neurol. 12, 201–208. doi: 10.3988/jcn.2016.12.2.201, 27074295 PMC4828567

[ref10] Ansari M. A. Scheff S. W. (2010). Oxidative stress in the progression of Alzheimer disease in the frontal cortex. J. Neuropathol. Exp. Neurol. 69, 155–167. doi: 10.1097/NEN.0b013e3181cb5af4, 20084018 PMC2826839

[ref11] Arvanitakis Z. Capuano A. W. Leurgans S. E. Bennett D. A. Schneider J. A. (2016). Relation of cerebral vessel disease to Alzheimer's disease dementia and cognitive function in elderly people: a cross-sectional study. Lancet Neurol. 15, 934–943. doi: 10.1016/S1474-4422(16)30029-1, 27312738 PMC4969105

[ref12] Asahi M. Wang X. Mori T. Sumii T. Jung J.-C. Moskowitz M. A. . (2001). Effects of matrix metalloproteinase-9 gene knock-out on the proteolysis of blood–brain barrier and white matter components after cerebral ischemia. J. Neurosci. 21, 7724–7732. doi: 10.1523/JNEUROSCI.21-19-07724.2001 11567062 PMC6762894

[ref13] Atucha E. Roozendaal B. (2015). The inhibitory avoidance discrimination task to investigate accuracy of memory. Front. Behav. Neurosci. 9:60. doi: 10.3389/fnbeh.2015.00060, 25814942 PMC4357306

[ref14] Badea A. Ali-Sharief A. A. Johnson G. A. (2007). Morphometric analysis of the C57BL/6J mouse brain. NeuroImage 37, 683–693. doi: 10.1016/j.neuroimage.2007.05.046, 17627846 PMC2176152

[ref15] Beauchet O. Annweiler C. Callisaya M. L. De Cock A.-M. Helbostad J. L. Kressig R. W. . (2016). Poor gait performance and prediction of dementia: results from a Meta-analysis. J. Am. Med. Dir. Assoc. 17, 482–490. doi: 10.1016/j.jamda.2015.12.092, 26852960 PMC5319598

[ref16] Bennett S. A. Tenniswood M. Chen J. H. Davidson C. M. Keyes M. T. Fortin T. . (1998). Chronic cerebral hypoperfusion elicits neuronal apoptosis and behavioral impairment. Neuroreport 9, 161–166. doi: 10.1097/00001756-199801050-00033, 9592069

[ref17] Bink D. I. Ritz K. Aronica E. Van Der Weerd L. Daemen M. J. (2013). Mouse models to study the effect of cardiovascular risk factors on brain structure and cognition. J. Cereb. Blood Flow Metab. 33, 1666–1684. doi: 10.1038/jcbfm.2013.140, 23963364 PMC3824184

[ref18] Bohnen N. I. Albin R. L. (2011). White matter lesions in Parkinson disease. Nat. Rev. Neurol. 7, 229–236. doi: 10.1038/nrneurol.2011.21, 21343896 PMC3739056

[ref19] Boltze J. Förschler A. Nitzsche B. Waldmin D. Hoffmann A. Boltze C. M. . (2008). Permanent middle cerebral artery occlusion in sheep: a novel large animal model of focal cerebral ischemia. J. Cereb. Blood Flow Metab. 28, 1951–1964. doi: 10.1038/jcbfm.2008.89, 18698332

[ref20] Bouhrara M. Reiter D. A. Bergeron C. M. Zukley L. M. Ferrucci L. Resnick S. M. . (2018). Evidence of demyelination in mild cognitive impairment and dementia using a direct and specific magnetic resonance imaging measure of myelin content. Alzheimers Dement. 14, 998–1004. doi: 10.1016/j.jalz.2018.03.007, 29679574 PMC6097903

[ref21] Cao Y. Gou Z. Du Y. Fan Y. Liang L. Yan Y. . (2016). Glutamatergic and central cholinergic dysfunction in the CA1, CA2 and CA3 fields on spatial learning and memory in chronic cerebral ischemia—induced vascular dementia of rats. Neurosci. Lett. 620, 169–176. doi: 10.1016/j.neulet.2016.03.039 27040427

[ref22] Cavanagh L. Paulsen J. S. (2024). Neuropsychology and vascular cognitive impairment and dementia. Neurol. Clin. 42, 809–820. doi: 10.1016/j.ncl.2024.05.006, 39343476 PMC12261353

[ref23] Chaudhari A. Padmar J. Awathale S. Goyal S. Nakhate K. Sherikar A. (2025). From glial cells to pain pathways: ICAM-1 as a central player in neuroinflammation and neuropathy. Discov. Neurosci. 20:5. doi: 10.1186/s13064-025-00201-0

[ref24] Chen L. Deng H. Cui H. Fang J. Zuo Z. Deng J. . (2018). Inflammatory responses and inflammation-associated diseases in organs. Oncotarget 9, 7204–7218. doi: 10.18632/oncotarget.23208, 29467962 PMC5805548

[ref25] Chen Z.-R. Huang J.-B. Yang S.-L. Hong F.-F. (2022). Role of cholinergic signaling in Alzheimer’s disease. Molecules 27:1816. doi: 10.3390/molecules27061816, 35335180 PMC8949236

[ref26] Chen S. T. Sultzer D. L. Hinkin C. H. Mahler M. E. Cummings J. L. (1998). Executive dysfunction in Alzheimer's disease. J. Neuropsychiatry Clin. Neurosci. 10, 426–432. doi: 10.1176/jnp.10.4.426, 9813788

[ref27] Cho K.-O. La H. O. Cho Y.-J. Sung K.-W. Kim S. Y. (2006). Minocycline attenuates white matter damage in a rat model of chronic cerebral hypoperfusion. J. Neurosci. Res. 83, 285–291. doi: 10.1002/jnr.20727, 16385583

[ref28] Choi B.-R. Kim D.-H. Back D. B. Kang C. H. Moon W.-J. Han J.-S. . (2016). Characterization of White matter injury in a rat model of chronic cerebral Hypoperfusion. Stroke 47, 542–547. doi: 10.1161/STROKEAHA.115.011679, 26670084

[ref29] Choi B.-R. Lee S. R. Han J.-S. Woo S.-K. Kim K. M. Choi D.-H. . (2011). Synergistic memory impairment through the interaction of chronic cerebral Hypoperfusion and Amlyloid toxicity in a rat model. Stroke 42, 2595–2604. doi: 10.1161/STROKEAHA.111.620179, 21737797

[ref30] Choo I. H. Carter S. F. Schöll M. L. Nordberg A. (2014). Astrocytosis measured by 11C-deprenyl PET correlates with decrease in gray matter density in the parahippocampus of prodromal Alzheimer’s patients. Eur. J. Nucl. Med. Mol. Imaging 41, 2120–2126. doi: 10.1007/s00259-014-2859-7, 25077930

[ref31] Chouliaras L. O’Brien J. T. (2023). The use of neuroimaging techniques in the early and differential diagnosis of dementia. Mol. Psychiatry 28, 4084–4097. doi: 10.1038/s41380-023-02215-8, 37608222 PMC10827668

[ref32] Choy M. Ganesan V. Thomas D. L. Thornton J. S. Proctor E. King M. D. . (2006). The chronic vascular and Haemodynamic response after permanent bilateral common carotid occlusion in newborn and adult rats. J. Cereb. Blood Flow Metab. 26, 1066–1075. doi: 10.1038/sj.jcbfm.9600259, 16395291

[ref33] Chung E.-H. Iwasaki K. Mishima K. Egashira N. Fujiwara M. (2002). Repeated cerebral ischemia induced hippocampal cell death and impairments of spatial cognition in the rat. Life Sci. 72, 609–619. doi: 10.1016/S0024-3205(02)02269-5, 12467902

[ref34] Clancy U. Kancheva A. K. Valdes Hernandez M. D. C. Jochems A. C. C. Munoz Maniega S. Quinn T. J. . (2024). Imaging biomarkers of VCI: a focused update. Stroke 55, 791–800. doi: 10.1161/STROKEAHA.123.044171, 38445496

[ref35] Colloby S. J. Firbank M. J. Pakrasi S. Perry E. K. Pimlott S. L. Wyper D. J. . (2011). Alterations in nicotinic α4β2 receptor binding in vascular dementia using 123I-5IA-85380 SPECT: comparison with regional cerebral blood flow. Neurobiol. Aging 32, 293–301. doi: 10.1016/j.neurobiolaging.2009.02.005, 19269714

[ref36] Corriveau R. A. Bosetti F. Emr M. Gladman J. T. Koenig J. I. Moy C. S. . (2016). The science of vascular contributions to cognitive impairment and dementia (VCID): a framework for advancing research priorities in the cerebrovascular biology of cognitive decline. Cell. Mol. Neurobiol. 36, 281–288. doi: 10.1007/s10571-016-0334-7, 27095366 PMC4859348

[ref37] Cummings J. L. (1987). Multi-infarct dementia: diagnosis and management. Psychosomatics 28, 117–126. doi: 10.1016/S0033-3182(87)72553-5, 3324157

[ref38] Daneshjoo S. Park J. Y. Moreno J. (2022). A mouse model of naturally occurring age-related cognitive impairment. Aging Pathobiol. Therapeut. 4, 87–89. doi: 10.31491/APT.2022.09.090, 36250162 PMC9562129

[ref39] de Bortoli V. C. Zangrossi Junior H. de Aguiar Correa F. M. Almeida Sde S. de Oliveira A. M. (2005). Inhibitory avoidance memory retention in the elevated T-maze is impaired after perivascular manipulation of the common carotid arteries. Life Sci. 76, 2103–2114. doi: 10.1016/j.lfs.2004.10.035, 15826877

[ref40] De La Torre J. C. Fortin T. (1994). A chronic physiological rat model of dementia. Behav. Brain Res. 63, 35–40. doi: 10.1016/0166-4328(94)90048-5, 7945975

[ref41] Debette S. Markus H. S. (2010). The clinical importance of white matter hyperintensities on brain magnetic resonance imaging: systematic review and meta-analysis. BMJ 341, c3666–c3666. doi: 10.1136/bmj.c3666, 20660506 PMC2910261

[ref42] Denic A. Macura S. I. Mishra P. Gamez J. D. Rodriguez M. Pirko I. (2011). MRI in rodent models of brain disorders. Neurotherapeutics 8, 3–18. doi: 10.1007/s13311-010-0002-4, 21274681 PMC3075741

[ref43] Du S.-Q. Wang X.-R. Xiao L.-Y. Tu J.-F. Zhu W. He T. . (2017). Molecular mechanisms of vascular dementia: what can be learned from animal models of chronic cerebral hypoperfusion? Mol. Neurobiol. 54, 3670–3682. doi: 10.1007/s12035-016-9915-1 27206432

[ref44] Duchowny K. A. Ackley S. F. Brenowitz W. D. Wang J. Zimmerman S. C. Caunca M. R. . (2022). Associations between handgrip strength and dementia risk, cognition, and neuroimaging outcomes in the UK biobank cohort study. JAMA Netw. Open 5:e2218314. doi: 10.1001/jamanetworkopen.2022.18314, 35737388 PMC9227006

[ref45] Duncombe J. Kitamura A. Hase Y. Ihara M. Raj K. N. Horsburgh K. (2017). Chronic cerebral hypoperfusion: a key mechanism leading to vascular cognitive impairment and dementia. Closing the translational gap between rodent models and human vascular cognitive impairment and dementia. Clin. Sci. 131, 2451–2468. doi: 10.1042/CS20160727 28963120

[ref46] Eklöf B. Siesjö B. K. (1972). The effect of bilateral carotid artery ligation upon the blood flow and the energy state of the rat brain. Acta Physiol. Scand. 86, 155–165. doi: 10.1111/j.1748-1716.1972.tb05322.x, 4640167

[ref47] Elhassanien M. E. M. El-Heneedy Y. A. E. Ramadan K. M. Kotait M. A. Elkholy A. Elhamrawy M. Y. . (2021). 'Gait and balance impairments in patients with subcortical vascular cognitive impairment', Egypt. J. Neurol. Psychiatr. Neurosurg 57:56. doi: 10.1186/s41983-021-00293-5

[ref48] Engelhardt E. Moreira D. M. Laks J. (2009). The brain subcortical white matter and aging: a quantitative fractional anisotropy analysis. Dement. Neuropsychol. 3, 228–233. doi: 10.1590/s1980-57642009dn30300009, 29213633 PMC5618978

[ref49] Erkinjuntti T. (2003). Subcortical ischemic vascular disease and dementia. Int. Psychogeriatr. 15, 23–26. doi: 10.1017/S1041610203008925, 16191213

[ref50] Fagerli E. Jackson C. W. Escobar I. Ferrier F. J. Lao E. J. P. Saul I. . (2024). Resveratrol mitigates cognitive impairments and cholinergic cell loss in the medial septum in a mouse model of gradual cerebral hypoperfusion. Antioxidants 13:984. doi: 10.3390/antiox13080984, 39199230 PMC11351397

[ref51] Farkas E. Donka G. De Vos R. A. I. Mihály A. Bari F. Luiten P. G. M. (2004a). Experimental cerebral hypoperfusion induces white matter injury and microglial activation in the rat brain. Acta Neuropathol. 108, 57–64. doi: 10.1007/s00401-004-0864-9, 15138777

[ref52] Farkas E. Institóris Á. Domoki F. Mihály A. Luiten P. G. M. Bari F. (2004b). Diazoxide and dimethyl sulphoxide prevent cerebral hypoperfusion-related learning dysfunction and brain damage after carotid artery occlusion. Brain Res. 1008, 252–260. doi: 10.1016/j.brainres.2004.02.037, 15145763

[ref53] Farkas E. Luiten P. G. M. Bari F. (2007). Permanent, bilateral common carotid artery occlusion in the rat: a model for chronic cerebral hypoperfusion-related neurodegenerative diseases. Brain Res. Rev. 54, 162–180. doi: 10.1016/j.brainresrev.2007.01.003, 17296232

[ref54] Ferguson E. L. Thoma M. Buto P. T. Wang J. Glymour M. M. Hoffmann T. J. . (2024). Visual impairment, eye conditions, and diagnoses of neurodegeneration and dementia. JAMA Netw. Open 7:e2424539. doi: 10.1001/jamanetworkopen.2024.24539, 39078629 PMC11289698

[ref058] Fifield K. E. Vanderluit J. L. (2020). Rapid degeneration of neurons in the penumbra region following a small, focal ischemic stroke. Eur J Neurosci. 52, 3196–3214. doi: 10.1111/ejn.14678 31945213

[ref55] Frank M. J. Loughry B. O'Reilly R. C. (2001). Interactions between frontal cortex and basal ganglia in working memory: a computational model. Cogn. Affect. Behav. Neurosci. 1, 137–160. doi: 10.3758/CABN.1.2.137, 12467110

[ref56] Gannon O. J. Robison L. S. Custozzo A. J. Zuloaga K. L. (2019). Sex differences in risk factors for vascular contributions to cognitive impairment & dementia. Neurochem. Int. 127, 38–55. doi: 10.1016/j.neuint.2018.11.014, 30471324

[ref57] Gold G. KöVari E. Herrmann F. O. R. Canuto A. Hof P. R. Michel J.-P. . (2005). Cognitive consequences of thalamic, basal ganglia, and deep White matter lacunes in brain aging and dementia. Stroke 36, 1184–1188. doi: 10.1161/01.STR.0000166052.89772.b5, 15891000

[ref58] Gooch J. Wilcock D. M. (2016). Animal models of vascular cognitive impairment and dementia (VCID). Cell. Mol. Neurobiol. 36, 233–239. doi: 10.1007/s10571-015-0286-3, 26988696 PMC11482509

[ref59] Gorter R. P. Baron W. (2020). Matrix metalloproteinases shape the oligodendrocyte (niche) during development and upon demyelination. Neurosci. Lett. 729:134980. doi: 10.1016/j.neulet.2020.134980, 32315713

[ref60] Goto K. Ishige A. Sekiguchi K. Iizuka S. Sugimoto A. Yuzurihara M. . (1990). Effects of cycloheximide on delayed neuronal death in rat hippocampus. Brain Res. 534, 299–302. doi: 10.1016/0006-8993(90)90144-Z, 2073592

[ref61] Gottfries C. G. Blennow K. Karlsson I. Wallin A. (1994). The neurochemistry of vascular dementia. Dement. Geriatr. Cogn. Disord. 5, 163–167. doi: 10.1159/000106715, 8087172

[ref62] Graeff F. G. Netto C. F. Jr H. Z. (1998). The elevated T-maze as an experimental model of anxiety. Neurosci. Biobehav. Rev. 23, 237–246. doi: 10.1016/S0149-7634(98)00024-4, 9884116

[ref63] Gregory S. Bridgeman K. Darwin H. Barbato M. Booi L. Brugulat Serrat A. . (2025). Associations of estrogen with modifiable and non-modifiable risk factors for dementia: a narrative review. Alzheimers Dement. 21:e70873. doi: 10.1002/alz.70873 41263327 PMC12631545

[ref64] Gregory L. J. O'Neill M. J. Nunn J. A. Gray J. A. Williams S. C. R. (2001). Diffusion-weighted magnetic resonance imaging detects early neuropathology following four vessel occlusion ischemia in the rat. J. Magn. Reson. Imaging 14, 207–214. doi: 10.1002/jmri.1175, 11536396

[ref65] Hainsworth A. H. Allan S. M. Boltze J. Cunningham C. Farris C. Head E. . (2017). Translational models for vascular cognitive impairment: a review including larger species. BMC Med. 15:16. doi: 10.1186/s12916-017-0793-9, 28118831 PMC5264492

[ref66] Hanse E. Seth H. Riebe I. (2013). AMPA-silent synapses in brain development and pathology. Nat. Rev. Neurosci. 14, 839–850. doi: 10.1038/nrn3642, 24201185

[ref67] Hartman R. E. Lee J. M. Zipfel G. J. Wozniak D. F. (2005). Characterizing learning deficits and hippocampal neuron loss following transient global cerebral ischemia in rats. Brain Res. 1043, 48–56. doi: 10.1016/j.brainres.2005.02.030, 15862517

[ref68] Hattori Y. Enmi J.-I. Iguchi S. Saito S. Yamamoto Y. Nagatsuka K. . (2016a). Substantial reduction of parenchymal cerebral blood flow in mice with bilateral common carotid artery stenosis. Sci. Rep. 6:32179. doi: 10.1038/srep32179, 27535801 PMC4989493

[ref69] Hattori Y. Enmi J.-I. Iguchi S. Saito S. Yamamoto Y. Tsuji M. . (2016b). Gradual carotid artery stenosis in mice closely replicates Hypoperfusive vascular dementia in humans. J. Am. Heart Assoc. 5:e002757. doi: 10.1161/JAHA.115.002757, 26903005 PMC4802480

[ref70] Hattori Y. Enmi J.-I. Kitamura A. Yamamoto Y. Saito S. Takahashi Y. . (2015). A novel mouse model of subcortical infarcts with dementia. J. Neurosci. 35, 3915–3928. doi: 10.1523/JNEUROSCI.3970-14.2015, 25740520 PMC6605574

[ref71] Hattori Y. Kitamura A. Nagatsuka K. Ihara M. (2014a). A novel mouse model of ischemic carotid artery disease. PLoS One 9:e100257. doi: 10.1371/journal.pone.0100257, 24940742 PMC4062537

[ref72] Hattori Y. Kitamura A. Tsuji M. Nagatsuka K. Ihara M. (2014b). Motor and cognitive impairment in a mouse model of ischemic carotid artery disease. Neurosci. Lett. 581, 1–6. doi: 10.1016/j.neulet.2014.08.009, 25123442

[ref73] Heim C. Zhang J. Lan J. Sieklucka M. Kurz T. Riederer P. . (2000). Cerebral oligaemia episode triggers free radical formation and late cognitive deficiencies. Eur. J. Neurosci. 12, 715–725. doi: 10.1046/j.1460-9568.2000.00916.x, 10712651

[ref74] Heiss W.-D. Rosenberg G. A. Thiel A. Berlot R. De Reuck J. (2016). Neuroimaging in vascular cognitive impairment: a state-of-the-art review. BMC Med. 14:174. doi: 10.1186/s12916-016-0725-0, 27806705 PMC5094143

[ref75] Hernandez A. R. Winesett S. P. Federico Q. P. Williams S. A. Burke S. N. Clark D. J. (2020). A cross-species model of dual-task walking in young and older humans and rats. Front. Aging Neurosci. 12:276. doi: 10.3389/fnagi.2020.00276, 32982717 PMC7492995

[ref76] Hietamies T. M. Ostrowski C. Pei Z. Feng L. McCabe C. Work L. M. . (2018). Variability of functional outcome measures used in animal models of stroke and vascular cognitive impairment – a review of contemporary studies. J. Cereb. Blood Flow Metab. 38, 1872–1884. doi: 10.1177/0271678X18799858, 30203705 PMC6259321

[ref77] Holland P. R. Searcy J. L. Salvadores N. Scullion G. Chen G. Lawson G. . (2015). Gliovascular disruption and cognitive deficits in a mouse model with features of small vessel disease. J. Cereb. Blood Flow Metab. 35, 1005–1014. doi: 10.1038/jcbfm.2015.12, 25669904 PMC4640247

[ref78] Huang L. Zhe-Bao W. Zhuge Q. Zheng W. Shao B. Wang B. . (2014). Glial scar formation occurs in the human brain after ischemic stroke. Int. J. Med. Sci. 11, 344–348. doi: 10.7150/ijms.8140, 24578611 PMC3936028

[ref79] Iadecola C. Duering M. Hachinski V. Joutel A. Pendlebury S. T. Schneider J. A. . (2019). Vascular cognitive impairment and dementia. J. Am. Coll. Cardiol. 73, 3326–3344. doi: 10.1016/j.jacc.2019.04.034, 31248555 PMC6719789

[ref80] Ihara M. Tomimoto H. (2011). Lessons from a mouse model characterizing features of vascular cognitive impairment with White matter changes. J. Aging Res. 2011, 1–11. doi: 10.4061/2011/978761, 22132331 PMC3216359

[ref81] Irie K. Mishima K. Ishibashi D. Egashira N. Iwasaki K. Fujiwara M. (2002). Involvement of bcl-family expression in the spatial memory impairment induced by repeated ischemia. Life Sci. 72, 621–629. doi: 10.1016/S0024-3205(02)02270-1, 12467903

[ref82] Ishikawa H. Shindo A. Mizutani A. Tomimoto H. Lo E. H. Arai K. (2023). A brief overview of a mouse model of cerebral hypoperfusion by bilateral carotid artery stenosis. J. Cereb. Blood Flow Metab. 43, 18–36. doi: 10.1177/0271678X231154597, 36883344 PMC10638994

[ref83] Iwasaki K. Kitamura Y. Ohgami Y. Mishima K. Fujiwara M. (1996). The disruption of spatial cognition and changes in brain amino acid, monoamine and acetylcholine in rats with transient cerebral ischemia. Brain Res. 709, 163–172. doi: 10.1016/0006-8993(95)01235-4, 8833752

[ref84] Jiang T. Zhang L. Pan X. Zheng H. Chen X. Li L. . (2017). Physical exercise improves cognitive function together with microglia phenotype modulation and remyelination in chronic cerebral hypoperfusion. Front. Cell. Neurosci. 11:404. doi: 10.3389/fncel.2017.00404, 29311834 PMC5743796

[ref85] Jiménez-Ruiz A. Aguilar-Fuentes V. Becerra-Aguiar N. N. Roque-Sanchez I. Ruiz-Sandoval J. L. (2024). Vascular cognitive impairment and dementia: a narrative review. Dement. Neuropsychol. 18:e20230116. doi: 10.1590/1980-5764-dn-2023-0116, 39318380 PMC11421556

[ref86] Jiwa N. S. Garrard P. Hainsworth A. H. (2010). Experimental models of vascular dementia and vascular cognitive impairment: a systematic review. J. Neurochem. 115, 814–828. doi: 10.1111/j.1471-4159.2010.06958.x, 20731763

[ref87] Johnson A. C. (2023). Hippocampal vascular supply and its role in vascular cognitive impairment. Stroke 54, 673–685. doi: 10.1161/STROKEAHA.122.038263, 36848422 PMC9991081

[ref88] Justić H. Barić A. Šimunić I. Radmilović M. Ister R. Škokić S. . (2022). Redefining the Koizumi model of mouse cerebral ischemia: a comparative longitudinal study of cerebral and retinal ischemia in the Koizumi and Longa middle cerebral artery occlusion models. J. Cereb. Blood Flow Metab. 42, 2080–2094. doi: 10.1177/0271678X221109873, 35748043 PMC9580169

[ref89] Kalantari S. Soltani M. Maghbooli M. Khoshe Mehr F. S. Kalantari Z. Borji S. . (2025). Cerebral blood flow alterations measured by ASL-MRI as a predictor of vascular dementia in small vessel ischemic disease. Radiología (English Edition) 67, 28–37. doi: 10.1016/j.rxeng.2024.03.013, 39978877

[ref90] Kalaria R. N. (2016). Neuropathological diagnosis of vascular cognitive impairment and vascular dementia with implications for Alzheimer’s disease. Acta Neuropathol. 131, 659–685. doi: 10.1007/s00401-016-1571-z, 27062261 PMC4835512

[ref91] Kawa H. Ahmed Z. Majid A. Chen R. (2025). Inhibition of matrix metalloproteinases to reduce blood brain barrier disruption and haemorrhagic transformation in ischaemic stroke: go broad or go narrow? Neuropharmacology 262:110192. doi: 10.1016/j.neuropharm.2024.110192, 39419277

[ref92] Khan R. Devlin P. Urayama A. Ritzel R. M. (2025). Models and mechanisms of post-stroke dementia and cognitive impairment. Front. Stroke 4:1563924. doi: 10.3389/fstro.2025.1563924, 40757092 PMC12315578

[ref93] Khan M. B. Hoda M. N. Vaibhav K. Giri S. Wang P. Waller J. L. . (2015). Remote ischemic Postconditioning: harnessing endogenous protection in a murine model of vascular cognitive impairment. Transl. Stroke Res. 6, 69–77. doi: 10.1007/s12975-014-0374-6, 25351177 PMC4297613

[ref94] Kim H. J. Jang H. Kim H. J. Na D. L. Yoon J. H. (2025). Kinematic characteristics in patients with subcortical vascular cognitive impairment: a quantitative analysis of digitized spiral drawing metrics. Sci. Rep. 15:3955. doi: 10.1038/s41598-025-88604-1, 39890926 PMC11785735

[ref95] Kim H. Urquhart R. Pontarelli F. Jover-Mengual T. Ofengeim D. Hwang J.-Y. (2023). Protocol for establishing a global ischemia model using a 4-vessel occlusion in rats. STAR Protocols 4:102630. doi: 10.1016/j.xpro.2023.102630, 38264871 PMC10751550

[ref96] Kimura S. Iwata M. Takase H. Lo E. H. Arai K. (2025). Oxidative stress and chronic cerebral hypoperfusion: an overview from preclinical rodent models. J. Cereb. Blood Flow Metab. 45, 381–395. doi: 10.1177/0271678X241305899, 39663901 PMC11635795

[ref97] Kirova A.-M. Bays R. B. Lagalwar S. (2015). Working memory and executive function decline across Normal aging, mild cognitive impairment, and Alzheimer’s disease. Biomed. Res. Int. 2015, 1–9. doi: 10.1155/2015/748212, 26550575 PMC4624908

[ref98] Kitamura A. Fujita Y. Oishi N. Kalaria R. N. Washida K. Maki T. . (2012). Selective white matter abnormalities in a novel rat model of vascular dementia. Neurobiol. Aging 33, 1012.e25–1012.e35. doi: 10.1016/j.neurobiolaging.2011.10.033, 22133276

[ref99] Kleinschnitz C. Fluri F. Schuhmann M. (2015). Animal models of ischemic stroke and their application in clinical research. Drug Des. Devel. Ther. 9:3445. doi: 10.2147/DDDT.S56071, 26170628 PMC4494187

[ref100] Kochan N. A. Breakspear M. Slavin M. J. Valenzuela M. McCraw S. Brodaty H. . (2010). Functional alterations in brain activation and deactivation in mild cognitive impairment in response to a graded working memory challenge. Dement. Geriatr. Cogn. Disord. 30, 553–568. doi: 10.1159/000322112, 21252551

[ref101] Krafft P. R. Bailey E. L. Lekic T. Rolland W. B. Altay O. Tang J. . (2012). Etiology of stroke and choice of models. Int. J. Stroke 7, 398–406. doi: 10.1111/j.1747-4949.2012.00838.x, 22712741 PMC6986354

[ref102] Kumaran D. (2008). Short-term memory and the human Hippocampus. J. Neurosci. 28, 3837–3838. doi: 10.1523/JNEUROSCI.0046-08.2008, 18400882 PMC6670459

[ref103] Kumral E. Deveci E. E. Erdoğan C. Enüstün C. (2015). Isolated hippocampal infarcts: vascular and neuropsychological findings. J. Neurol. Sci. 356, 83–89. doi: 10.1016/j.jns.2015.06.011, 26142022

[ref104] Lai T. W. Zhang S. Wang Y. T. (2014). Excitotoxicity and stroke: identifying novel targets for neuroprotection. Prog. Neurobiol. 115, 157–188. doi: 10.1016/j.pneurobio.2013.11.006, 24361499

[ref105] Lakhan S. E. Kirchgessner A. Tepper D. Leonard A. (2013). Matrix Metalloproteinases and blood-brain barrier disruption in acute ischemic stroke. Front. Neurol. 4:32. doi: 10.3389/fneur.2013.00032, 23565108 PMC3615191

[ref106] Lassmann H. (2014). Mechanisms of white matter damage in multiple sclerosis. Glia 62, 1816–1830. doi: 10.1002/glia.22597, 24470325

[ref107] Lecordier S. Manrique-Castano D. El Moghrabi Y. Elali A. (2021). Neurovascular alterations in vascular dementia: emphasis on risk factors. Front. Aging Neurosci. 13:727590. doi: 10.3389/fnagi.2021.727590, 34566627 PMC8461067

[ref108] Lee R. M. K. W. (1995). Morphology of cerebral arteries. Pharmacol. Ther. 66, 149–173. doi: 10.1016/0163-7258(94)00071-A, 7630927

[ref109] Lee K. M. Bang J. H. Han J.-S. Kim B. Y. Lee I. S. Kang H. W. . (2013). Cardiotonic pill attenuates white matter and hippocampal damage via inhibiting microglial activation and downregulating ERK and p38 MAPK signaling in chronic cerebral hypoperfused rat. BMC Complement. Altern. Med. 13:334. doi: 10.1186/1472-6882-13-334, 24274593 PMC4222777

[ref110] Lee N. K. Kim H. Yang J. Kim J. Son J. P. Jang H. . (2020). Heterogeneous disease progression in a mouse model of vascular cognitive impairment. Int. J. Mol. Sci. 21:2820. doi: 10.3390/ijms21082820, 32316637 PMC7215687

[ref111] Lee S.-R. Tsuji K. Lee S.-R. Eng H. L. (2004). Role of matrix Metalloproteinases in delayed neuronal damage after transient global cerebral ischemia. J. Neurosci. 24, 671–678. doi: 10.1523/JNEUROSCI.4243-03.2004, 14736853 PMC6729252

[ref112] Lennon M. J. Sachdev P. S. (2026). Vascular cognitive impairment and dementia: prevention, treatments, mechanisms and management options for the future. Neuropsychopharmacology. doi: 10.1038/s41386-026-02331-3 [E-pub ahead of print], 41554956

[ref113] León-Moreno L. C. Castañeda-Arellano R. Rivas-Carrillo J. D. Dueñas-Jiménez S. H. (2020). Challenges and improvements of developing an ischemia mouse model through bilateral common carotid artery occlusion. J. Stroke Cerebrovasc. Dis. 29:104773. doi: 10.1016/j.jstrokecerebrovasdis.2020.104773, 32199775

[ref114] Li X. Li D. Li Q. Li Y. Li K. Li S. . (2016). Hippocampal subfield volumetry in patients with subcortical vascular mild cognitive impairment. Sci. Rep. 6:20873. doi: 10.1038/srep20873, 26876151 PMC4753487

[ref115] Li M. Meng N. Guo X. Niu X. Zhao Z. Wang W. . (2020). Dl-3-n-butylphthalide promotes remyelination and suppresses inflammation by regulating AMPK/SIRT1 and STAT3/NF-κB signaling in chronic cerebral hypoperfusion. Front. Aging Neurosci. 12:137. doi: 10.3389/fnagi.2020.00137, 32581761 PMC7296049

[ref116] Li Y. Shuping Zhu L. Yuan H. L. Li H. Tong S. (2013). Predicting the ischemic infarct volume at the first minute after occlusion in rodent stroke model by laser speckle imaging of cerebral blood flow. J. Biomed. Opt. 18:076024. doi: 10.1117/1.JBO.18.7.076024, 23887483

[ref117] Li Y. Tan L. Yang C. He L. Liu L. Deng B. . (2023). Distinctions between the Koizumi and Zea Longa methods for middle cerebral artery occlusion (MCAO) model: a systematic review and meta-analysis of rodent data. Sci. Rep. 13:10247. doi: 10.1038/s41598-023-37187-w, 37353569 PMC10290095

[ref118] Li J. Zhang L. Chu Y. Namaka M. Deng B. Kong J. . (2016). Astrocytes in oligodendrocyte lineage development and White matter pathology. Front. Cell. Neurosci. 10:119. doi: 10.3389/fncel.2016.00119, 27242432 PMC4861901

[ref119] Li H.-W. Zhang L. Qin C. (2019). Current state of research on non-human primate models of Alzheimer’s disease. Anim. Models Exp. Med. 2, 227–238. doi: 10.1002/ame2.12092, 31942555 PMC6930996

[ref120] Li N. Zhiqiang G. Li Y. Xiaojie F. Wang J. Bai H. (2015). A modified bilateral carotid artery stenosis procedure to develop a chronic cerebral hypoperfusion rat model with an increased survival rate. J. Neurosci. Methods 255, 115–121. doi: 10.1016/j.jneumeth.2015.08.002, 26277419

[ref121] Lin K. A. Doraiswamy P. M. (2015). When Mars versus Venus is not a cliché: gender differences in the neurobiology of Alzheimer’s disease. Front. Neurol. 5:288. doi: 10.3389/fneur.2014.00288 25628598 PMC4290582

[ref122] Lituma P. J. Woo E. O’Hara B. F. Castillo P. E. Sibinga N. E. S. Nandi S. (2021). Altered synaptic connectivity and brain function in mice lacking microglial adapter protein Iba1. Proc. Natl. Acad. Sci. 118:e2115539118. doi: 10.1073/pnas.2115539118, 34764226 PMC8609554

[ref123] Liu L. Hou J. Cui S. Zhao X. Liu Z. Longenecker J. C. . (2026). Sex differences in the association of vascular risk and APOE genotype with cognitive decline and dementia: evidence from a U.S. longitudinal study. Lancet Reg. Health 54:101346. doi: 10.1016/j.lana.2025.101346, 41536502 PMC12796570

[ref124] Longa E. Z. Weinstein P. R. Carlson S. Cummins R. (1989). Reversible middle cerebral artery occlusion without craniectomy in rats. Stroke 20, 84–91. doi: 10.1161/01.STR.20.1.84, 2643202

[ref125] Lourenço C. F. Ledo A. Dias C. Barbosa R. M. Laranjinha J. (2015). Neurovascular and neurometabolic derailment in aging and Alzheimer's disease. Front. Aging Neurosci. 7:103. doi: 10.3389/fnagi.2015.00103 26074816 PMC4445047

[ref126] Lowry E. Puthusseryppady V. Johnen A.-K. Renoult L. Hornberger M. (2021). Cognitive and neuroimaging markers for preclinical vascular cognitive impairment. Cereb. Circul. Cogn. Behav. 2:100029. doi: 10.1016/j.cccb.2021.100029, 36324708 PMC9616378

[ref127] Lueg G. Gross C. C. Lohmann H. Johnen A. Kemmling A. Deppe M. . (2015). Clinical relevance of specific T-cell activation in the blood and cerebrospinal fluid of patients with mild Alzheimer's disease. Neurobiol. Aging 36, 81–89. doi: 10.1016/j.neurobiolaging.2014.08.008, 25277040

[ref128] Ma Y. Chen S. Li Y. Wang J. Yang J. Jing J. . (2023). Effects of dl-3-n-butylphthalide on cognitive functions and blood–brain barrier in chronic cerebral hypoperfusion rats. Naunyn Schmiedeberg's Arch. Pharmacol. 396, 3207–3220. doi: 10.1007/s00210-023-02530-5, 37243759 PMC10567816

[ref129] Madigan J. B. Wilcock D. M. Hainsworth A. H. (2016). Vascular contributions to cognitive impairment and dementia. Stroke 47, 1953–1959. doi: 10.1161/STROKEAHA.116.012066, 27301939 PMC4927375

[ref130] Mahinrad S. Sorond F. Gorelick P. B. (2023). The role of vascular risk factors in cognitive impairment and dementia and prospects for prevention. Clin. Geriatr. Med. 39, 123–134. doi: 10.1016/j.cger.2022.07.007, 36404025 PMC11806923

[ref131] Maki T. Ihara M. Fujita Y. Nambu T. Miyashita K. Yamada M. . (2011). Angiogenic and Vasoprotective effects of Adrenomedullin on prevention of cognitive decline after chronic cerebral Hypoperfusion in mice. Stroke 42, 1122–1128. doi: 10.1161/STROKEAHA.110.603399, 21393586

[ref132] Marquié M. Castilla-Martí M. Valero S. Martínez J. Sánchez D. Hernández I. . (2019). Visual impairment in aging and cognitive decline: experience in a memory clinic. Sci. Rep. 9:8698. doi: 10.1038/s41598-019-45055-9, 31213626 PMC6581941

[ref133] Mather M. Clewett D. Sakaki M. Harley C. W. (2016). Norepinephrine ignites local hotspots of neuronal excitation: how arousal amplifies selectivity in perception and memory. Behav. Brain Sci. 39, 1–100. doi: 10.1017/S0140525X15000667 PMC5830137 26126507

[ref134] Matute C. Alberdi E. Domercq M. Sánchez-Gómez M.-V. Pérez-Samartín A. Rodríguez-Antigüedad A. . (2007). Excitotoxic damage to white matter. J. Anat. 210, 693–702. doi: 10.1111/j.1469-7580.2007.00733.x, 17504270 PMC2375761

[ref135] Maurer S. V. Williams C. L. (2017). The cholinergic system modulates memory and hippocampal plasticity via its interactions with non-neuronal cells. Front. Immunol. 8:1489. doi: 10.3389/fimmu.2017.01489, 29167670 PMC5682336

[ref136] McIver S. R. Muccigrosso M. Gonzales E. R. Lee J. M. Roberts M. S. Sands M. S. . (2010). Oligodendrocyte degeneration and recovery after focal cerebral ischemia. Neuroscience 169, 1364–1375. doi: 10.1016/j.neuroscience.2010.04.070, 20621643 PMC3789594

[ref137] McVeigh C. Passmore P. (2006). Vascular dementia: prevention and treatment. Clin. Interv. Aging 1, 229–235. doi: 10.2147/ciia.2006.1.3.229, 18046875 PMC2695177

[ref138] Mehla J. Lacoursiere S. Stuart E. McDonald R. J. Mohajerani M. H. (2017). Gradual cerebral Hypoperfusion impairs fear conditioning and object recognition learning and memory in mice: potential roles of neurodegeneration and cholinergic dysfunction. J. Alzheimer's Dis 61, 283–293. doi: 10.3233/JAD-170635, 29154281

[ref139] Mervosh N. Devi G. (2025). Estrogen, menopause, and Alzheimer’s disease: understanding the link to cognitive decline in women. Front. Mol. Biosci. 12:1634302. doi: 10.3389/fmolb.2025.1634302, 40661313 PMC12256231

[ref140] Mijajlović M. D. Pavlović A. Brainin M. Heiss W.-D. Quinn T. J. Ihle-Hansen H. B. . (2017). Post-stroke dementia – a comprehensive review. BMC Med. 15:11. doi: 10.1186/s12916-017-0779-7, 28095900 PMC5241961

[ref141] Mok V. (2023). Neuroimaging biomarkers in vascular dementia. J. Neurol. Sci. 455:120937. doi: 10.1016/j.jns.2023.120937

[ref142] Mokhber N. Shariatzadeh A. Avan A. Saber H. Babaei G. S. Chaimowitz G. . (2021). Cerebral blood flow changes during aging process and in cognitive disorders: a review. Neuroradiol. J. 34, 300–307. doi: 10.1177/19714009211002778, 33749402 PMC8447819

[ref143] Montero-Odasso M. Verghese J. Beauchet O. Hausdorff J. M. (2012). Gait and cognition: a complementary approach to understanding brain function and the risk of falling. J. Am. Geriatr. Soc. 60, 2127–2136. doi: 10.1111/j.1532-5415.2012.04209.x, 23110433 PMC3498517

[ref144] Moretti R. Torre P. Antonello R. M. Esposito F. Bellini G. (2011). Gait and equilibrium in subcortical vascular dementia. Curr. Gerontol. Geriatr. Res. 2011, 1–7. doi: 10.1155/2011/263507, 21547149 PMC3085296

[ref145] Neto C. J. Ferreira B. Paganelli R. A. Benetoli A. Lima K. C. M. Milani H. (2005). Permanent, 3-stage, 4-vessel occlusion as a model of chronic and progressive brain hypoperfusion in rats: a neurohistological and behavioral analysis. Behav. Brain Res. 160, 312–322. doi: 10.1016/j.bbr.2004.12.016, 15863227

[ref146] Nguyen D. H. Thomas Cunningham J. Sumien N. (2021). Estrogen receptor involvement in vascular cognitive impairment and vascular dementia pathogenesis and treatment. GeroScience 43, 159–166. doi: 10.1007/s11357-020-00263-4, 32902819 PMC8050128

[ref147] Ni J.-W. Matsumoto K. Li H.-B. Murakami Y. Watanabe H. (1995). Neuronal damage and decrease of central acetylcholine level following permanent occlusion of bilateral common carotid arteries in rat. Brain Res. 673, 290–296. doi: 10.1016/0006-8993(94)01436-L, 7606443

[ref148] Nishio K. Ihara M. Yamasaki N. Kalaria R. N. Maki T. Fujita Y. . (2010). A mouse model characterizing features of vascular dementia with hippocampal atrophy. Stroke 41, 1278–1284. doi: 10.1161/STROKEAHA.110.581686, 20448204

[ref149] Nordberg A. (2001). Nicotinic receptor abnormalities of Alzheimer’s disease: therapeutic implications. Biol. Psychiatry 49, 200–210. doi: 10.1016/S0006-3223(00)01125-2, 11230871

[ref150] Ohtsuki S. Sato S. Yamaguchi H. Kamoi M. Asashima T. Terasaki T. (2007). Exogenous expression of claudin-5 induces barrier properties in cultured rat brain capillary endothelial cells. J. Cell. Physiol. 210, 81–86. doi: 10.1002/jcp.20823, 16998798

[ref151] Onken M. Berger S. Kristian T. (2012). Simple model of forebrain ischemia in mouse. J. Neurosci. Methods 204, 254–261. doi: 10.1016/j.jneumeth.2011.11.022, 22146544 PMC3273657

[ref152] Pantoni L. Garcia J. H. Gutierrez J. A. (1996). Cerebral White matter is highly vulnerable to ischemia. Stroke 27, 1641–1647. doi: 10.1161/01.STR.27.9.1641, 8784142

[ref153] Patel A. Moalem A. Cheng H. Babadjouni R. M. Patel K. Hodis D. M. . (2017). Chronic cerebral hypoperfusion induced by bilateral carotid artery stenosis causes selective recognition impairment in adult mice. Neurol. Res. 39, 910–917. doi: 10.1080/01616412.2017.1355423, 28828966 PMC5591078

[ref154] Pereira M. Francine E. D. Ferreira F. De Oliveira R. M. W. Milani H. (2012). Time-course of neurodegeneration and memory impairment following the 4-vessel occlusion/internal carotid artery model of chronic cerebral hypoperfusion in middle-aged rats. Behav. Brain Res. 229, 340–348. doi: 10.1016/j.bbr.2012.01.023, 22274621

[ref155] Perez F. I. Rivera V. M. Meyer J. S. Gay J. R. Taylor R. L. Mathew N. T. (1975). Analysis of intellectual and cognitive performance in patients with multi-infarct dementia, vertebrobasilar insufficiency with dementia, and Alzheimer's disease. J. Neurol. Neurosurg. Psychiatry 38, 533–540. doi: 10.1136/jnnp.38.6.533, 1151420 PMC492023

[ref156] Perosa V. Priester A. Ziegler G. Cardenas-Blanco A. Dobisch L. Spallazzi M. . (2020). Hippocampal vascular reserve associated with cognitive performance and hippocampal volume. Brain 143, 622–634. doi: 10.1093/brain/awz383, 31994699 PMC7009470

[ref157] Perry E. K. Gibson P. H. Blessed G. Perry R. H. Tomlinson B. E. (1977). Neurotransmitter enzyme abnormalities in senile dementia. J. Neurol. Sci. 34, 247–265. doi: 10.1016/0022-510X(77)90073-9, 144789

[ref158] Perry E. Ziabreva I. Perry R. Aarsland D. Ballard C. (2005). Absence of cholinergic deficits in “pure” vascular dementia. Neurology 64, 132–133. doi: 10.1212/01.WNL.0000148591.63727.80, 15642917

[ref159] Petito C. K. Olarte J.-P. Roberts B. Nowak T. S. Pulsinell W. A. (1998). Selective glial vulnerability following transient global ischemia in rat brain. J. Neuropathol. Exp. Neurol. 57, 231–238. doi: 10.1097/00005072-199803000-00004, 9600215

[ref160] Picciotto M. R. Higley M. J. Mineur Y. S. (2012). Acetylcholine as a neuromodulator: cholinergic signaling shapes nervous system function and behavior. Neuron 76, 116–129. doi: 10.1016/j.neuron.2012.08.036 23040810 PMC3466476

[ref161] Piskorowski R. A. Chevaleyre V. (2023). Hippocampal area CA2: interneuron disfunction during pathological states. Front. Neural Circuits 17:1181032. doi: 10.3389/fncir.2023.1181032, 37180763 PMC10174260

[ref162] Plaschke K. Bardenheuer H. J. Martin E. Sartor K. Heiland S. (2006). Evolution of apparent diffusion coefficient and transverse relaxation time (T2) in the subchronic stage of global cerebral oligemia in different rat models. Exp. Brain Res. 169, 361–368. doi: 10.1007/s00221-005-0146-3, 16328309

[ref163] Plaschke K. Grant M. Weigand M. A. Züchner J. Martin E. Bardenheuer H. J. (2001). Neuromodulatory effect of propentofylline on rat brain under acute and long-term hypoperfusion. Br. J. Pharmacol. 133, 107–116. doi: 10.1038/sj.bjp.0704061, 11325800 PMC1572772

[ref164] Pulsinelli W. A. Brierley J. B. (1979). A new model of bilateral hemispheric ischemia in the unanesthetized rat. Stroke 10, 267–272. doi: 10.1161/01.STR.10.3.267, 37614

[ref165] Quintana D. D. Ren X. Heng H. Engler-Chiurazzi E. B. Rellick S. L. Lewis S. E. . (2018). Gradual common carotid artery occlusion as a novel model for cerebrovascular Hypoperfusion. Metab. Brain Dis. 33, 2039–2044. doi: 10.1007/s11011-018-0312-5, 30267298 PMC6342504

[ref166] Rajeev V. Fann D. Y. Dinh Q. N. Kim H. A. Michael De Silva T. Lai M. K. P. . (2022). Pathophysiology of blood brain barrier dysfunction during chronic cerebral hypoperfusion in vascular cognitive impairment. Theranostics 12, 1639–1658. doi: 10.7150/thno.68304, 35198062 PMC8825579

[ref167] Raji C. A. Benzinger T. L. S. (2022). The value of neuroimaging in dementia diagnosis. Continuum 28, 800–821. doi: 10.1212/CON.0000000000001133, 35678403 PMC9993425

[ref168] Ribitsch I. Baptista P. M. Lange-Consiglio A. Melotti L. Patruno M. Jenner F. . (2020). Large animal models in regenerative medicine and tissue engineering: to do or not to do. Front. Bioeng. Biotechnol. 8:972. doi: 10.3389/fbioe.2020.00972, 32903631 PMC7438731

[ref169] Rodríguez J. J. Olabarria M. Chvatal A. Verkhratsky A. (2009). Astroglia in dementia and Alzheimer's disease. Cell Death Differentiat. 16, 378–385. doi: 10.1038/cdd.2008.172, 19057621

[ref170] Roh J. H. Lee J.-H. (2014). Recent updates on subcortical ischemic vascular dementia. J. Stroke 16, 18–26. doi: 10.5853/jos.2014.16.1.18, 24741561 PMC3961819

[ref171] Román G. C. Erkinjuntti T. Wallin A. Pantoni L. Chui H. C. (2002). Subcortical ischaemic vascular dementia. Lancet Neurol. 1, 426–436. doi: 10.1016/S1474-4422(02)00190-4, 12849365

[ref172] Román G. C. Kalaria R. N. (2006). Vascular determinants of cholinergic deficits in Alzheimer disease and vascular dementia. Neurobiol. Aging 27, 1769–1785. doi: 10.1016/j.neurobiolaging.2005.10.004, 16300856

[ref173] Rosenberg G. A. Sullivan N. Esiri M. M. (2001). White matter damage is associated with matrix Metalloproteinases in vascular dementia. Stroke 32, 1162–1168. doi: 10.1161/01.STR.32.5.1162, 11340226

[ref174] Rundek T. Tolea M. Ariko T. Fagerli E. A. Camargo C. J. (2022). Vascular Cognitive Impairment (VCI). Neurotherapeutics 19, 68–88. doi: 10.1007/s13311-021-01170-y, 34939171 PMC9130444

[ref175] Sakurai K. Li H. Inamura N. Masuoka N. Hisatsune T. (2020). Relationship between elevated impulsivity and cognitive declines in elderly community-dwelling individuals. Sci. Rep. 10:21032. doi: 10.1038/s41598-020-78124-5, 33273585 PMC7713053

[ref176] Santisteban M. M. Iadecola C. (2025). The pathobiology of neurovascular aging. Neuron 113, 49–70. doi: 10.1016/j.neuron.2024.12.014, 39788087 PMC12136575

[ref177] Sarhan M. Wohlfeld C. Perry-Mills A. Meyers J. Fadel J. Murphy E. A. . (2025). The pathophysiology of mixed Alzheimer's disease and vascular dementia. Theranostics 15, 9793–9818. doi: 10.7150/thno.118737 41041058 PMC12486426

[ref178] Sarti C. Pantoni L. Bartolini L. Inzitari D. (2002). Persistent impairment of gait performances and working memory after bilateral common carotid artery occlusion in the adult Wistar rat. Behav. Brain Res. 136, 13–20. doi: 10.1016/S0166-4328(02)00090-6, 12385786

[ref179] Schetters S. T. T. Gomez-Nicola D. Garcia-Vallejo J. J. Van Kooyk Y. (2018). Neuroinflammation: microglia and T cells get ready to tango. Front. Immunol. 8:1905. doi: 10.3389/fimmu.2017.01905, 29422891 PMC5788906

[ref180] Schmidt-Kastner R. Aguirre-Chen C. Saul I. Yick L. Hamasaki D. Busto R. . (2005). Astrocytes react to oligemia in the forebrain induced by chronic bilateral common carotid artery occlusion in rats. Brain Res. 1052, 28–39. doi: 10.1016/j.brainres.2005.06.018, 16023090

[ref181] Schmidt-Kastner R. Paschen W. Ophoff B. G. Hossmann K. A. (1989). A modified four-vessel occlusion model for inducing incomplete forebrain ischemia in rats. Stroke 20, 938–946. doi: 10.1161/01.STR.20.7.938, 2749852

[ref182] Schmidt-Kastner R. Truettner J. Lin B. Zhao W. Saul I. Busto R. . (2001). Transient changes of brain-derived neurotrophic factor (BDNF) mRNA expression in hippocampus during moderate ischemia induced by chronic bilateral common carotid artery occlusions in the rat. Mol. Brain Res. 92, 157–166. doi: 10.1016/s0169-328x(01)00157-7, 11483252

[ref183] Schröder H. Moser N. Huggenberger S. (2020). The Mouse Circle of Willis. Cham, Switzerland: Springer International Publishing.

[ref184] Schuff N. Matsumoto S. Kmiecik J. Studholme C. Antao D. Ezekiel F. . (2009). Cerebral blood flow in ischemic vascular dementia and Alzheimer's disease, measured by arterial spin-labeling magnetic resonance imaging. Alzheimers Dement. 5, 454–462. doi: 10.1016/j.jalz.2009.04.1233, 19896584 PMC2802181

[ref185] Shibata M. Ohtani R. Ihara M. Tomimoto H. (2004). White matter lesions and glial activation in a novel mouse model of chronic cerebral Hypoperfusion. Stroke 35, 2598–2603. doi: 10.1161/01.STR.0000143725.19053.60, 15472111

[ref186] Shibata M. Yamasaki N. Miyakawa T. Kalaria R. N. Fujita Y. Ohtani R. . (2007). Selective impairment of working memory in a mouse model of chronic cerebral Hypoperfusion. Stroke 38, 2826–2832. doi: 10.1161/STROKEAHA.107.490151, 17761909

[ref187] Simchick G. Scheulin K. M. Sun W. Sneed S. E. Fagan M. M. Cheek S. R. . (2021). Detecting functional connectivity disruptions in a translational pediatric traumatic brain injury porcine model using resting-state and task-based fMRI. Sci. Rep. 11:12406. doi: 10.1038/s41598-021-91853-5, 34117318 PMC8196021

[ref188] Simchick G. Shen A. Campbell B. Park H. J. West F. D. Zhao Q. (2019). Pig brains have homologous resting-state networks with human brains. Brain Connect. 9, 566–579. doi: 10.1089/brain.2019.0673, 31115245 PMC6727477

[ref189] Skrobot O. A. Black S. E. Chen C. Decarli C. Erkinjuntti T. Ford G. A. . (2018, 2018). Progress toward standardized diagnosis of vascular cognitive impairment: guidelines from the vascular impairment of cognition classification consensus study. Alzheimers Dement. 14, 280–292. doi: 10.1016/j.jalz.2017.09.007, 29055812

[ref190] Skrobot O. A. O'Brien J. Black S. Chen C. Decarli C. Erkinjuntti T. . (2017, 2017). The vascular impairment of cognition classification consensus study. Alzheimers Dement. 13, 624–633. doi: 10.1016/j.jalz.2016.10.007, 27960092

[ref191] Smith J. A. Das A. Ray S. K. Banik N. L. (2012). Role of pro-inflammatory cytokines released from microglia in neurodegenerative diseases. Brain Res. Bull. 87, 10–20. doi: 10.1016/j.brainresbull.2011.10.004, 22024597 PMC9827422

[ref192] Song J. Kim Y.-S. Lee D. H. Lee S. H. Park H. J. Lee D. . (2019). Neuroprotective effects of oleic acid in rodent models of cerebral ischaemia. Sci. Rep. 9:10732. doi: 10.1038/s41598-019-47057-z, 31341184 PMC6656890

[ref193] Sood R. Yang Y. Taheri S. Candelario-Jalil E. Estrada E. Y. Walker E. J. . (2009). Increased apparent diffusion coefficients on MRI linked with matrix Metalloproteinases and edema in White matter after bilateral carotid artery occlusion in rats. J. Cereb. Blood Flow Metab. 29, 308–316. doi: 10.1038/jcbfm.2008.121, 18941468

[ref194] Sopala M. Danysz W. (2001). Chronic cerebral hypoperfusion in the rat enhances age-related deficits in spatial memory. J. Neural Transm. 108, 1445–1456. doi: 10.1007/s007020100019, 11810407

[ref195] Soria G. Tudela R. Márquez-Martín A. Camón L. Batalle D. Muñoz-Moreno E. . (2013). The ins and outs of the BCCAo model for chronic Hypoperfusion: a multimodal and longitudinal MRI approach. PLoS One 8:e74631. doi: 10.1371/journal.pone.0074631, 24058609 PMC3776744

[ref196] Staines D. R. Brenu E. W. Marshall-Gradisnik S. (2008). Postulated role of vasoactive neuropeptide-related immunopathology of the blood brain barrier and Virchow-Robin spaces in the Aetiology of neurological-related conditions. Mediat. Inflamm. 2008, 1–5. doi: 10.1155/2008/792428, 19229345 PMC2643053

[ref197] Stewart C. R. Stringer M. S. Shi Y. Thrippleton M. J. Wardlaw J. M. (2021). Associations between White matter Hyperintensity burden, cerebral blood flow and transit time in small vessel disease: an updated Meta-analysis. Front. Neurol. 12:647848. doi: 10.3389/fneur.2021.647848, 34017302 PMC8129542

[ref198] Sudduth T. L. Weekman E. M. Price B. R. Gooch J. L. Woolums A. Norris C. M. . (2017). Time-course of glial changes in the hyperhomocysteinemia model of vascular cognitive impairment and dementia (VCID). Neuroscience 341, 42–51. doi: 10.1016/j.neuroscience.2016.11.024, 27890830 PMC5911565

[ref199] Sugawara T. T. Fujimura M. Morita-Fujimura Y. Kawase M. Chan P. H. (1999). T mitochondrial release of cytochromecCorresponds to the selective vulnerability of hippocampal CA1 neurons in rats after transient global cerebral ischemia. J. Neurosci. 19, RC39–RC39. 10559429 10.1523/JNEUROSCI.19-22-j0002.1999 PMC6782966

[ref200] Sultzer D. L. Levin H. S. Mahler M. E. High W. M. Cummings J. L. (1993). A comparison of psychiatric symptoms in vascular dementia and Alzheimer's disease. Am. J. Psychiatry 150, 1806–1812. doi: 10.1176/ajp.150.12.1806, 8238634

[ref0202] Sun W. Chen Y. Zhang Y. Geng Y. Tang X. Guo R. . (2021). A modified four vessel occlusion model of global cerebral ischemia in rats. J. Neurosci. Methods 352: 109090. doi: 10.1016/j.jneumeth.2021.109090 33516736

[ref201] Sweeney M. D. Sagare A. P. Zlokovic B. V. (2018). Blood–brain barrier breakdown in Alzheimer disease and other neurodegenerative disorders. Nat. Rev. Neurol. 14, 133–150. doi: 10.1038/nrneurol.2017.188, 29377008 PMC5829048

[ref202] Taguchi A. Kasahara Y. Nakagomi T. Stern D. M. Fukunaga M. Ishikawa M. . (2010). A reproducible and simple model of permanent cerebral ischemia in CB-17 and SCID mice. J. Exp. Stroke Transl. Med. 3, 28–33. doi: 10.6030/1939-067X-3.1.28, 20865060 PMC2943401

[ref203] Tambo W. Powell K. Wadolowski S. Unadkat P. Chang E. H. Ledoux C. . (2025). Vasoactive neuropeptide dysregulation: a novel mechanism of microvascular dysfunction in vascular cognitive impairment. Alzheimers Dement. 21:e70925. doi: 10.1002/alz.70925, 41268782 PMC12635772

[ref204] Tanaka K.-I. Ogawa N. Asanuma M. Kondo Y. Nomura M. (1996). Relationship between cholinergic dysfunction and discrimination learning disabilities in Wistar rats following chronic cerebral hypoperfusion. Brain Res. 729, 55–65. doi: 10.1016/0006-8993(96)00400-3, 8874876

[ref205] Tao X. He J. Zhang Y. Yin Y. Yang C. Shang Y. . (2025). Fluid biomarkers of vascular cognitive impairment: from vascular pathophysiology to potential clinical applications. Neuroscience 579, 267–283. doi: 10.1016/j.neuroscience.2025.06.018, 40499808

[ref206] Teleanu R. I. Niculescu A. G. Roza E. Vladacenco O. Grumezescu A. M. Teleanu D. M. (2022). Neurotransmitters-key factors in neurological and neurodegenerative disorders of the central nervous system. Int. J. Mol. Sci. 23:5954. doi: 10.3390/ijms23115954, 35682631 PMC9180936

[ref207] Temedda M. N. Garnier-Crussard A. Moutet C. Mouchoux C. Dauphinot V. (2025). Comorbidities and comorbidity burden in patients with neurocognitive disorders: findings from the MEMORA cohort study. Eur. Geriatr. Med. 16, 2153–2167. doi: 10.1007/s41999-025-01288-8, 40866782 PMC12743666

[ref208] Ter Telgte A. Van Leijsen E. M. C. Wiegertjes K. Klijn C. J. M. Tuladhar A. M. De Leeuw F.-E. (2018). Cerebral small vessel disease: from a focal to a global perspective. Nat. Rev. Neurol. 14, 387–398. doi: 10.1038/s41582-018-0014-y 29802354

[ref209] Tian Z. Ji X. Liu J. (2022). Neuroinflammation in vascular cognitive impairment and dementia: current evidence, advances, and prospects. Int. J. Mol. Sci. 23:6224. doi: 10.3390/ijms23116224, 35682903 PMC9181710

[ref210] Tisato V. Rimondi E. Brombo G. Volpato S. Zurlo A. Zauli G. . (2016). Serum soluble tumor necrosis factor-related apoptosis-inducing ligand levels in older subjects with dementia and mild cognitive impairment. Dement. Geriatr. Cogn. Disord. 41, 273–280. doi: 10.1159/000446275, 27304551

[ref211] Tohgi H. Abe T. Kimura M. Saheki M. Takahashi S. (1996). Cerebrospinal fluid acetylcholine and choline in vascular dementia of Binswanger and multiple small infarct types as compared with Alzheimer-type dementia. J. Neural Transm. 103, 1211–1220. doi: 10.1007/BF01271206, 9013408

[ref212] Tomimoto H. Lin J.-X. Matsuo A. Ihara M. Ohtani R. Shibata M. . (2004). Different mechanisms of corpus callosum atrophy in Alzheimer?S disease and vascular dementia. J. Neurol. 251, 398–406. doi: 10.1007/s00415-004-0330-6, 15083283

[ref213] Toyama K. Koibuchi N. Uekawa K. Hasegawa Y. Kataoka K. Katayama T. . (2014). Apoptosis signal–regulating kinase 1 is a novel target molecule for cognitive impairment induced by chronic cerebral hypoperfusion. Arterioscler. Thromb. Vasc. Biol. 34, 616–625. doi: 10.1161/ATVBAHA.113.302440 24371084

[ref214] Tukacs V. Mittli D. Györffy B. A. Hunyady-Gulyás É. Hlatky D. Tóth V. . (2020). Chronic stepwise cerebral hypoperfusion differentially induces synaptic proteome changes in the frontal cortex, occipital cortex, and hippocampus in rats. Sci. Rep. 10:15999. doi: 10.1038/s41598-020-72868-w, 32994510 PMC7524772

[ref215] Tuo Q.-Z. Zou J.-J. Lei P. (2021). Rodent models of vascular cognitive impairment. J. Mol. Neurosci. 71, 1–12. doi: 10.1007/s12031-020-01733-2, 33107013

[ref216] Ueno K. I. Togashi H. Mori K. Matsumoto M. Ohashi S. Hoshino A. . (2002). Behavioural and pharmacological relevance of stroke-prone spontaneously hypertensive rats as an animal model of a developmental disorder. Behav. Pharmacol. 13, 1–13. doi: 10.1097/00008877-200202000-00001, 11990715

[ref217] Unekawa M. Tsukada N. Takizawa T. Tomita Y. Nakahara J. Izawa Y. (2024). Striatal blood flow changes by middle cerebral artery occlusion and its effect on neurological deficits in mice. Microcirculation 31:e12861. doi: 10.1111/micc.12861, 38762881

[ref218] Van De Pol L. Gertz H.-J. Scheltens P. Wolf H. (2011). Hippocampal atrophy in subcortical vascular dementia. Neurodegener Dis 8, 465–469. doi: 10.1159/000326695 21613775

[ref219] Van Der Flier W. M. Skoog I. Schneider J. A. Pantoni L. Mok V. Chen C. L. H. . (2018). Vascular cognitive impairment. Nat. Rev. Dis. Primers 4:18003. doi: 10.1038/nrdp.2018.3, 29446769

[ref220] Van Straaten E. C. W. Scheltens P. Barkhof F. (2004). MRI and CT in the diagnosis of vascular dementia. J. Neurol. Sci. 226, 9–12. doi: 10.1016/j.jns.2004.09.003, 15537511

[ref221] Verkhratsky A. Olabarria M. Noristani H. N. Yeh C.-Y. Rodriguez J. J. (2010). Astrocytes in Alzheimer's disease. Neurotherapeutics 7, 399–412. doi: 10.1016/j.nurt.2010.05.017, 20880504 PMC5084302

[ref222] Wahul A. B. Joshi P. C. Kumar A. Chakravarty S. (2018). Transient global cerebral ischemia differentially affects cortex, striatum and hippocampus in bilateral common carotid arterial occlusion (BCCAo) mouse model. J. Chem. Neuroanat. 92, 1–15. doi: 10.1016/j.jchemneu.2018.04.006, 29702163

[ref223] Wakita H. Tomimoto H. Akiguchi I. Kimura J. (1994). Glial activation and white matter changes in the rat brain induced by chronic cerebral hypoperfusion: an immunohistochemical study. Acta Neuropathol. 87, 484–492. doi: 10.1007/BF00294175, 8059601

[ref224] Walker E. J. Rosenberg G. A. (2010). Divergent role for MMP-2 in myelin breakdown and oligodendrocyte death following transient global ischemia. J. Neurosci. Res. 88, 764–773. doi: 10.1002/jnr.22257, 19830840 PMC4950938

[ref225] Waller S. B. Ball M. J. Reynolds M. A. London E. D. (1986). Muscarinic binding and choline acetyltransferase in postmortem brains of demented patients. Can. J. Neurol. Sci. 13, 528–532. doi: 10.1017/S0317167100037252, 3791067

[ref226] Wang Z. Fan J. Wang J. Li Y. Duan D. Guo D. . (2016). Chronic cerebral hypoperfusion induces long-lasting cognitive deficits accompanied by long-term hippocampal silent synapses increase in rats. Behav. Brain Res. 301, 243–252. doi: 10.1016/j.bbr.2015.12.047, 26756439

[ref227] Wang Y. Liu G. Hong D. Chen F. Ji X. Cao G. (2016). White matter injury in ischemic stroke. Prog. Neurobiol. 141, 45–60. doi: 10.1016/j.pneurobio.2016.04.005, 27090751 PMC5677601

[ref228] Wang J. Yang C. Wang H. Li D. Li T. Sun Y. . (2020). A new rat model of chronic cerebral hypoperfusion resulting in early-stage vascular cognitive impairment. Front. Aging Neurosci. 12:86. doi: 10.3389/fnagi.2020.00086, 32351379 PMC7174718

[ref229] Wang J. Zhang H.-Y. Tang X.-C. (2009). Cholinergic deficiency involved in vascular dementia: possible mechanism and strategy of treatment. Acta Pharmacol. Sin. 30, 879–888. doi: 10.1038/aps.2009.82, 19574993 PMC4006646

[ref230] Wang J. Zhang Y. Tian N. Ya D. Yang J. Jiang Y. . (2024). Mechanisms of glutamate metabolic function and dysfunction in vascular dementia. Neuroprotection 2, 33–48. doi: 10.1002/nep3.32, 41383444 PMC12486949

[ref231] Washida K. Hattori Y. Ihara M. (2019). Animal models of chronic cerebral Hypoperfusion: from mouse to primate. Int. J. Mol. Sci. 20:6176. doi: 10.3390/ijms20246176, 31817864 PMC6941004

[ref232] Wei G. Kibler K. K. Koehler R. C. Maruyama T. Narumiya S. Doré S. (2008). Prostacyclin receptor deletion aggravates hippocampal neuronal loss after bilateral common carotid artery occlusion in mouse. Neuroscience 156, 1111–1117. doi: 10.1016/j.neuroscience.2008.07.073, 18790018 PMC6010173

[ref233] Wei B. Wang Z. Wu S. Orgah J. Zhu J. Song W. (2021). Improving collateral circulation: a potential adjunctive strategy to prevent or slow the progression of vascular dementia. Neuropsychiatr. Dis. Treat. 17, 3061–3067. doi: 10.2147/NDT.S328446 34675517 PMC8502063

[ref234] Wells A. J. Vink R. Blumbergs P. C. Brophy B. P. Helps S. C. Knox S. J. . (2012). A surgical model of permanent and transient middle cerebral artery stroke in the sheep. PLoS One 7:e42157. doi: 10.1371/journal.pone.0042157, 22848737 PMC3407087

[ref235] Wendell C. R. Waldstein S. R. Ferrucci L. O’Brien R. J. Strait J. B. Zonderman A. B. (2012). Carotid atherosclerosis and prospective risk of dementia. Stroke 43, 3319–3324. doi: 10.1161/STROKEAHA.112.672527, 23103489 PMC3508298

[ref236] Weng Z. Cao C. Stepicheva N. A. Chen F. Foley L. M. Cao S. . (2023). A novel needle mouse model of vascular cognitive impairment and dementia. J. Neurosci. 43, 7351–7360. doi: 10.1523/JNEUROSCI.0282-23.2023, 37684030 PMC10621771

[ref237] Wharton S. B. Simpson J. E. Brayne C. Ince P. G. (2015). Age-associated white matter lesions: the MRC cognitive function and ageing study. Brain Pathol. 25, 35–43. doi: 10.1111/bpa.12219, 25521175 PMC8029351

[ref238] Wood Alexander M. Paterson J. Arvanitakis Z. Black S. E. Casaletto K. B. Christakis M. K. . (2025). Cardiovascular contributions to dementia: examining sex differences and female-specific factors. Alzheimers Dement. 21:e70610. doi: 10.1002/alz.70610, 40851413 PMC12375877

[ref239] World Health Organization (2025). “Dementia” fact sheet. Available online at: https://www.who.int/news-room/fact-sheets/detail/dementia (Accessed March 31, 2025).

[ref240] Xiang X. Wind K. Wiedemann T. Blume T. Shi Y. Briel N. . (2021). Microglial activation states drive glucose uptake and FDG-PET alterations in neurodegenerative diseases. Sci. Transl. Med. 13:eabe5640. doi: 10.1126/scitranslmed.abe5640, 34644146

[ref241] Xie S. Zhang Z. Chang F. Wang Y. Zhang Z. Zhou Z. . (2016). Subcortical White matter changes with Normal aging detected by multi-shot High resolution diffusion tensor imaging. PLoS One 11:e0157533. doi: 10.1371/journal.pone.0157533, 27332713 PMC4917173

[ref242] Xing Y. Wei C. Chu C. Zhou A. Li F. Wu L. . (2012). Stage-specific gender differences in cognitive and neuropsychiatric manifestations of vascular dementia. Am. J. Alzheimers Dis. Other Dement. 27, 433–438. doi: 10.1177/1533317512454712, 22930700 PMC10697341

[ref243] Xu W. Bai Q. Dong Q. Guo M. Cui M. (2022). Blood–brain barrier dysfunction and the potential mechanisms in chronic cerebral Hypoperfusion induced cognitive impairment. Front. Cell. Neurosci. 16:870674. doi: 10.3389/fncel.2022.870674, 35783093 PMC9243657

[ref244] Yagita Y. (2024). Distinct pathophysiology of small vessel disease from atherosclerosis. Hypertens. Res. 47, 3073–3074. doi: 10.1038/s41440-024-01890-6, 39294457

[ref245] Yamaguchi M. Calvert J. W. Kusaka G. Zhang J. H. (2005). One-stage anterior approach for four-vessel occlusion in rat. Stroke 36, 2212–2214. doi: 10.1161/01.STR.0000182238.08510.c5, 16166575

[ref246] Yamauchi H. Fukuyama H. Harada K. Nabatame H. Ogawa M. Ouchi Y. . (1993). Callosal atrophy parallels decreased cortical oxygen metabolism and neuropsychological impairment in Alzheimer's disease. Arch. Neurol. 50, 1070–1074. doi: 10.1001/archneur.1993.00540100061017, 8215966

[ref247] Yang H. M. (2025). Vascular dementia: from pathophysiology to therapeutic Frontiers. J. Clin. Med. 14:6611. doi: 10.3390/jcm14186611, 41010812 PMC12470695

[ref248] Yang Y. Estrada E. Y. Thompson J. F. Liu W. Rosenberg G. A. (2007). Matrix metalloproteinase-mediated disruption of tight junction proteins in cerebral vessels is reversed by synthetic matrix metalloproteinase inhibitor in focal ischemia in rat. J. Cereb. Blood Flow Metab. 27, 697–709. doi: 10.1038/sj.jcbfm.9600375, 16850029

[ref249] Yang Y. Kimura-Ohba S. Thompson J. Rosenberg G. A. (2016). Rodent models of vascular cognitive impairment. Transl. Stroke Res. 7, 407–414. doi: 10.1007/s12975-016-0486-2, 27498679 PMC5016244

[ref250] Yang Y. Ma K. Li S. Xiong T. (2025). Multifaceted role of nitric oxide in vascular dementia. Med. Gas Res. 15, 496–506. doi: 10.4103/mgr.MEDGASRES-D-24-00158, 40300885 PMC12124705

[ref251] Yang L. Song J. Nan D. Wan Y. Guo H. (2022). Cognitive impairments and blood-brain barrier damage in a mouse model of chronic cerebral Hypoperfusion. Neurochem. Res. 47, 3817–3828. doi: 10.1007/s11064-022-03799-3, 36308621 PMC9718874

[ref252] Yang L. Zhang D. Zhang Q. (2023). Astrocyte-mediated myelin phagocytosis in ischemia. Neurosci. Bull. 39, 167–169. doi: 10.1007/s12264-022-00917-7, 35829984 PMC9849496

[ref253] Yılmaz H. Bayraktutan U. (2025). Cerebral small vessel disease: therapeutic approaches targeting Neuroinflammation, oxidative stress, and endothelial dysfunction. Curr. Issues Mol. Biol. 47:232. doi: 10.3390/cimb47040232, 40699631 PMC12025420

[ref254] Zarow C. Vinters H. V. Ellis W. G. Weiner M. W. Mungas D. White L. . (2005). Correlates of hippocampal neuron number in Alzheimer's disease and ischemic vascular dementia. Ann. Neurol. 57, 896–903. doi: 10.1002/ana.20503, 15929035 PMC1851673

[ref255] Zhai Y. Yamashita T. Nakano Y. Sun Z. Shang J. Feng T. . (2016). Chronic cerebral Hypoperfusion accelerates Alzheimer’s disease pathology with cerebrovascular remodeling in a novel mouse model. J. Alzheimer's Dis 53, 893–905. doi: 10.3233/JAD-160345, 27314529

[ref256] Zhang Y. Bhavnani B. R. (2006). Glutamate-induced apoptosis in neuronal cells is mediated via caspase-dependent and independent mechanisms involving calpain and caspase-3 proteases as well as apoptosis inducing factor (AIF) and this process is inhibited by equine estrogens. BMC Neurosci. 7:49. doi: 10.1186/1471-2202-7-49, 16776830 PMC1526740

[ref257] Zhang X. Chen Z. Xiong Y. Zhou Q. Zhu L.-Q. Liu D. (2025). The emerging role of nitric oxide in the synaptic dysfunction of vascular dementia. Neural Regen. Res. 20, 402–415. doi: 10.4103/NRR.NRR-D-23-01353, 38819044 PMC11317957

[ref258] Zhang S. Lachance B. B. Mattson M. P. Jia X. (2021). Glucose metabolic crosstalk and regulation in brain function and diseases. Prog. Neurobiol. 204:102089. doi: 10.1016/j.pneurobio.2021.102089, 34118354 PMC8380002

[ref259] Zhang Q. Yan X. Du J. Chen Z. Chang C. (2022). Diffusion tensor imaging as a tool to evaluate the cognitive function of patients with vascular dementia. Neurologist 28, 143–149. doi: 10.1097/nrl.0000000000000461, 35986673 PMC10158599

[ref260] Zhao R.-R. Fei X. Xiao-Chen X. Tan G.-J. Liu L.-M. Ning W. . (2015). Effects of alpha-lipoic acid on spatial learning and memory, oxidative stress, and central cholinergic system in a rat model of vascular dementia. Neurosci. Lett. 587, 113–119. doi: 10.1016/j.neulet.2014.12.037, 25534501

[ref261] Zhao J. Li Q. Meng L. Wang F. Li Q. Yang F. . (2022). Relationship between MMP-9 serum levels and tHcy levels and total imaging load and cognitive dysfunction. J. Stroke Cerebrovasc. Dis. 31:106759. doi: 10.1016/j.jstrokecerebrovasdis.2022.106759, 36201989

[ref0258] Zhao Y. Zhang X. Chen X. Wei Y. (2022). Neuronal injuries in cerebral infarction and ischemic stroke: From mechanisms to treatment (Review). Int. J. Mol. Med. 49:15. doi: 10.3892/ijmm.2021.5070 34878154 PMC8711586

[ref262] Zheng C. Zeng R. Wu G. Hu Y. Yu H. (2024). Beyond vision: a view from eye to Alzheimer's disease and dementia. J. Prev Alzheimers Dis. 11, 469–483. doi: 10.14283/jpad.2023.118 38374754

[ref263] Zhou W. Xia S. Wang C. Yang Q. Verkhratsky A. Niu J. (2025). Critical analysis of translational potential of rodent models of white matter pathology across a wide spectrum of human diseases. Cell Death Dis. 16:580. doi: 10.1038/s41419-025-07893-6, 40744926 PMC12313980

[ref264] Zotin Z. Clara M. Sveikata L. Viswanathan A. Yilmaz P. (2021). Cerebral small vessel disease and vascular cognitive impairment: from diagnosis to management. Curr. Opin. Neurol. 34, 246–257. doi: 10.1097/WCO.0000000000000913, 33630769 PMC7984766

